# Classification of white blood cells (leucocytes) from blood smear imagery using machine and deep learning models: A global scoping review

**DOI:** 10.1371/journal.pone.0292026

**Published:** 2024-06-17

**Authors:** Rabia Asghar, Sanjay Kumar, Arslan Shaukat, Paul Hynds

**Affiliations:** 1 Spatiotemporal Environmental Epidemiology Research (STEER) Group, Technological University Dublin, Dublin, Ireland; 2 National University of Sciences and Technology (NUST), Islamabad, Pakistan; National Library of Medicine, UNITED STATES

## Abstract

Machine learning (ML) and deep learning (DL) models are being increasingly employed for medical imagery analyses, with both approaches used to enhance the accuracy of classification/prediction in the diagnoses of various cancers, tumors and bloodborne diseases. To date however, no review of these techniques and their application(s) within the domain of white blood cell (WBC) classification in blood smear images has been undertaken, representing a notable knowledge gap with respect to model selection and comparison. Accordingly, the current study sought to comprehensively identify, explore and contrast ML and DL methods for classifying WBCs. Following development and implementation of a formalized review protocol, a cohort of 136 primary studies published between January 2006 and May 2023 were identified from the global literature, with the most widely used techniques and best-performing WBC classification methods subsequently ascertained. Studies derived from 26 countries, with highest numbers from high-income countries including the United States (n = 32) and The Netherlands (n = 26). While WBC classification was originally rooted in conventional ML, there has been a notable shift toward the use of DL, and particularly convolutional neural networks (CNN), with 54.4% of identified studies (n = 74) including the use of CNNs, and particularly in concurrence with larger datasets and bespoke features e.g., parallel data pre-processing, feature selection, and extraction. While some conventional ML models achieved up to 99% accuracy, accuracy was shown to decrease in concurrence with decreasing dataset size. Deep learning models exhibited improved performance for more extensive datasets and exhibited higher levels of accuracy in concurrence with increasingly large datasets. Availability of appropriate datasets remains a primary challenge, potentially resolvable using data augmentation techniques. Moreover, medical training of computer science researchers is recommended to improve current understanding of leucocyte structure and subsequent selection of appropriate classification models. Likewise, it is critical that future health professionals be made aware of the power, efficacy, precision and applicability of computer science, soft computing and artificial intelligence contributions to medicine, and particularly in areas like medical imaging.

## 1. Introduction

White blood cells (WBCs) play a vital role in the human immune system. They identify and neutralize pathogens including bacteria, viruses, and cancer cells. Classification of WBCs is therefore vital for accurate and early diagnosis and treatment of a range of diseases and medical conditions [1]. Machine learning techniques, both traditional and deep, have been widely adopted for myriad applications, including medical image analysis (MIA). MIA is critical in modern healthcare systems, aiding medical professionals in making well-informed decisions. It is currently used to diagnose brain tumors, lung cancer, anemia, leukemia, and malaria, via a range of image modalities including Magnetic Resonance Imaging (MRI), Computed Tomography (CT-Scans), Ultrasounds, Positron Emission Tomography (PET), Blood Smear images, and hybrid modalities [2]. Accordingly, MIA has attracted significant attention from computer vision experts, with traditional and deep machine learning techniques having been applied in leukocyte segmentation, cancer detection, classification, medical image annotation, and image retrieval in computer-aided diagnosis (CAD). The efficacy of these methods therefore directly influences clinical diagnosis and treatment strategies, highlighting the significance of technological advancements, such as high-speed computational resources and improved hardware and storage capabilities for CAD [3–5]. One of the primary application areas for CAD systems using traditional machine learning and deep learning is segmentation and classification of leukocytes (WBCs). Leukocytes provide valuable information to medical professionals (doctors, hematologists, pathologists, and radiologists), for diagnosing various blood-related issues, including Human Immunodeficiency Virus (HIV) and blood cancer (leukemia). Changes in the WBC count and/or morphological cell alterations, for instance variations in size, shape, and color observed in blood smear images, can provide valuable insights into various health disorders [6–9].

**Fig 1:**
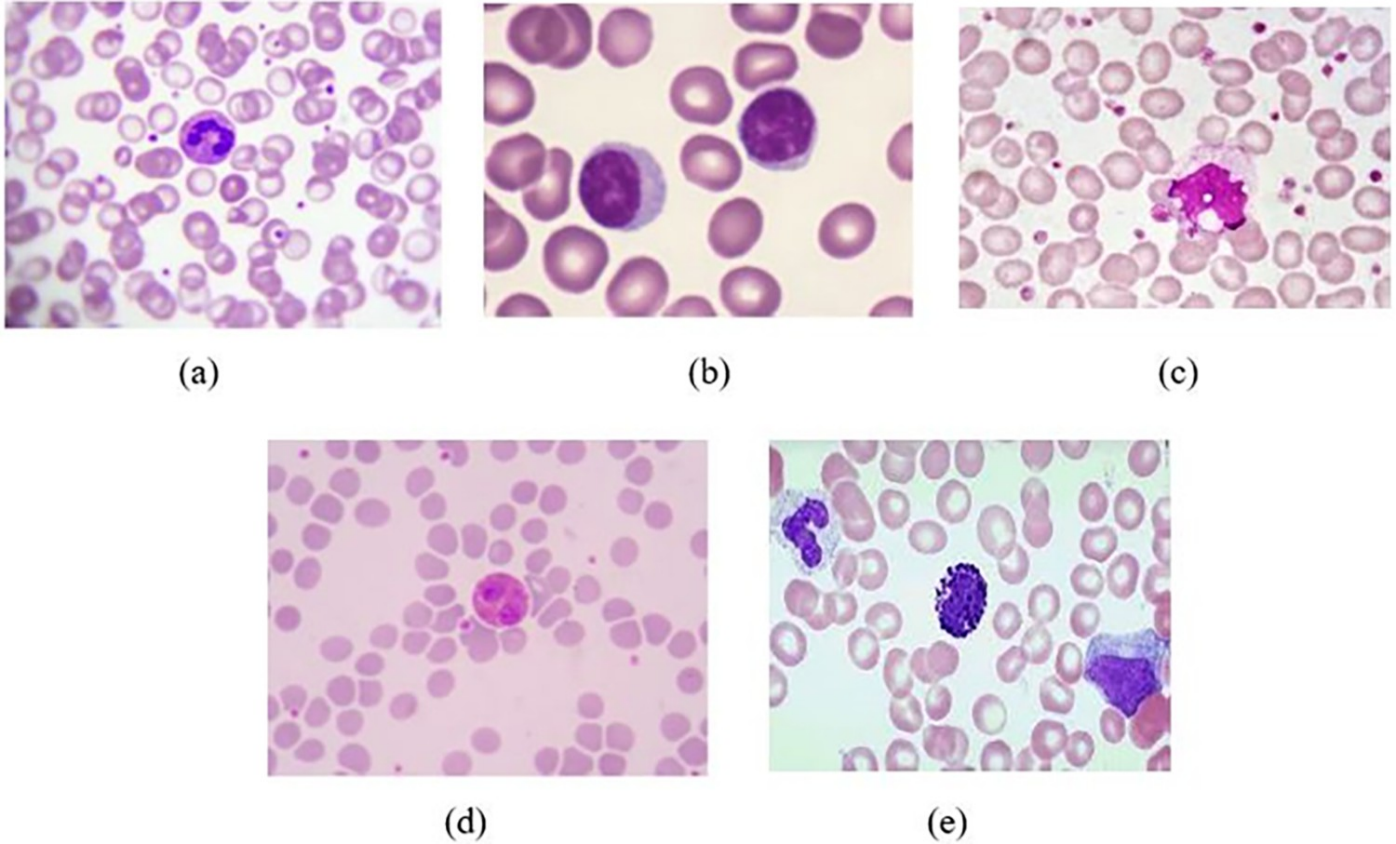
White blood cell categories [From 17] (a) Neutrophils (b) Lymphocytes (c) Monocytes (d) Eosinophils (e) Basophils.

**Fig 2:**
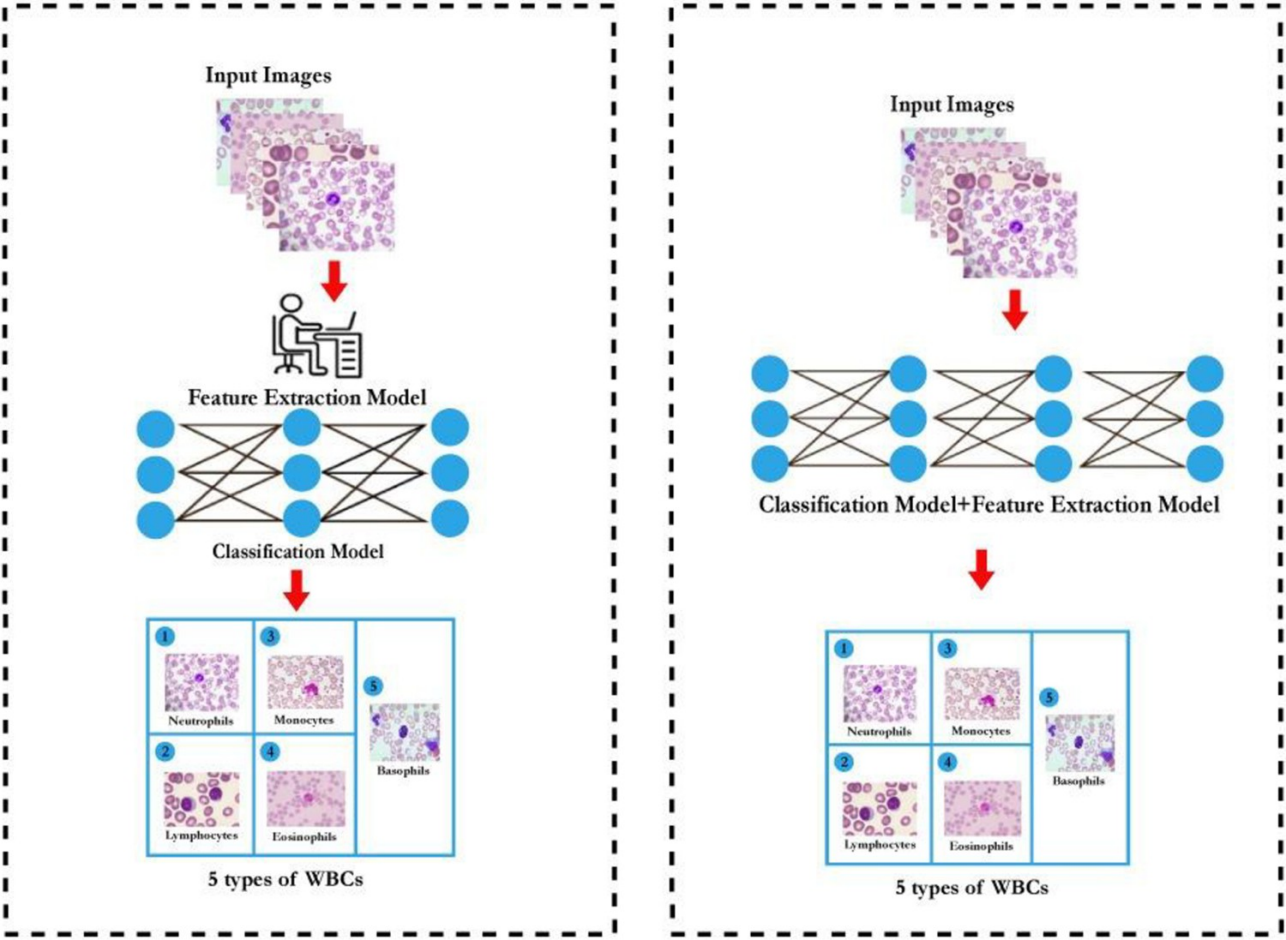
Neutrophil classification in blood smear images via (a) Traditional machine learning model (b) Advanced deep learning model.

Blood cells are categorized into three major types: WBCs (leukocytes), red blood cells (erythrocytes), and platelets (thrombocytes). Leukocytes are subdivided into five types: monocytes, lymphocytes, neutrophils, basophils, and eosinophils (Fig 1). Over the past two decades, significant advances have been made in traditional ML and DL methods for classification and segmentation of WBCs in microscopic blood smear images. Conventional methods depend on manually analyzing these images using microscopy, which is typically slow, laborious and error prone. Thus, development of automated and computer-aided systems has become crucial in accurate, systematic, unbiased and rapid clinical diagnosis and effective treatment. Automated analysis of WBCs in blood smear images can significantly reduce the workload of hematologists and provide fast, accurate, and efficient results to assist medical professionals in the diagnostic process [10–13]. There are two overarching methods typically used to achieve automated WBC classification in blood smear images: traditional machine learning (ML) and deep learning (DL) techniques. These techniques have the potential to make medical hematology more efficient. A generalized overview of machine learning and deep learning techniques used to classify WBCs is presented in Fig 2. The traditional machine learning process involves interconnected steps such as segmenting the region of interest and extracting features, followed by optimal classification [14, 15]. The feature extraction phase in traditional machine learning methods is challenging and directly impacts classification performance. More recently, deep learning approaches are increasingly used due to higher performance and decreasing complexity. Advanced deep learning methods with transfer learning have further improved implementation of automated systems for classification of WBCs. Notwithstanding the importance of ML and DL in medical image analysis (MIA), a gap remains in white blood cell classification via blood smear imagery; to date, no global review of these approaches is available in the published scientific literature. Accordingly, the present study sought to comprehensively identify and synthesize ML and DL methods, focusing on classifying five white blood cell types, and present this in concurrence with an overview of recommended future work, challenges and limitations associated with the identified approaches.

## 2. Review protocol

A well-organized and formally structured review process is essential to identify, scan, include/exclude and synthesize targeted literature which satisfies preexisting search criteria and effectively employs existing resources [16]. In the current review, the authors sought to incorporate the most recent and relevant research articles based on manual and automatic searches to identify all significant content. The approach was initiated by identifying pertinent research questions. The two research questions (RQ) formulated in accordance with the PCC (Population, Concept, Context) search framework are as follows:

- *How have systems been developed for classification of WBCs based on ML and DL*?
- *What are the applications of traditional machine learning and deep learning methods for effectively classifying WBCs in blood smear images*?

Relevant studies were identified using specific keywords extracted from the research questions (Table 1). These keywords covered various aspects, including segmentation, classification and detection of WBCs. The study explored machine learning techniques, involving both traditional and advanced deep learning methods. The research recognized the importance of big data and employed artificial intelligence (AI), as indicated by keywords like "Big data" and "Artificial Intelligence" respectively. This careful selection of keywords ensured a focused and comprehensive search across databases, resulting in retrieving relevant data for the study.

**Table 1:** Keywords used for iterative database searches.

**Blood Cells**			
Leukocyte(s) classification	A1	White blood cell(s) detection	A2
White blood cell(s) classification	A3	White blood cell(s) segmentation	A4
**Machine Learning**			
Machine learning	B1	Deep learning	B2
Big data	B3	Artificial Intelligence	B4

The next review phase after RQ development was identification of relevant articles/studies via automated searching of electronic databases based on extracted keywords from RQs (Iterative combinations of ((A1 –A4) x (B1 –B4)) from Table 1). Articles published from 2006 to May 2023 were included for review. To align with the study’s emphasis on recent research trends and technological progress, articles prior to 2006 were omitted. Research articles were located from three repositories including Google Scholar, Scopus, and Web of Science. The inclusion and exclusion criteria are presented in Table 2.

**Table 2:** Inclusion and exclusion criteria delineated via the PCC (Population, Concept, Context) search framework.

Inclusion	Exclusion
**Population** • Primary (peer-reviewed) original research articles • Participants/cohorts with accessible medical imagery (e.g., blood smear images, MRI, X-ray).	**Population** • Academic reviews (including all review typologies), book chapter(s), theses, conference presentations/abstracts, editorials. • Excluding individuals/imagery with lymphoma and myeloma due to potential impacts on white blood cell classification.
**Concept** • Studies specifically addressing classification of WBCs from human input data (See Contextual inclusion criteria below)	**Concept** • Studies not focused on white blood cell classification. For example: General health trends, patient demographics, or broader health outcomes without a central focus on white blood cell classification.
**Context** **Language**: Articles written in English. **Study period**: Research published between 2006 and May 2023. • Research conducted in the domain of medical image analysis (MIA). • Studies utilizing imaging modalities, including blood smear images, MRI, X-rays. • Research applying machine and deep learning techniques for white blood cell classification. For example: SVM, Navie Bayes, Decision Tree, ANN, CNN, Alexnet, ResNet family, GoogleNet etc.	**Context** **Language**: Articles not written in English **Study period**: Research published prior to January 1st 2006. • Studies conducted outside the scope of medical image analysis. For example vision transformers

**Fig 3:**
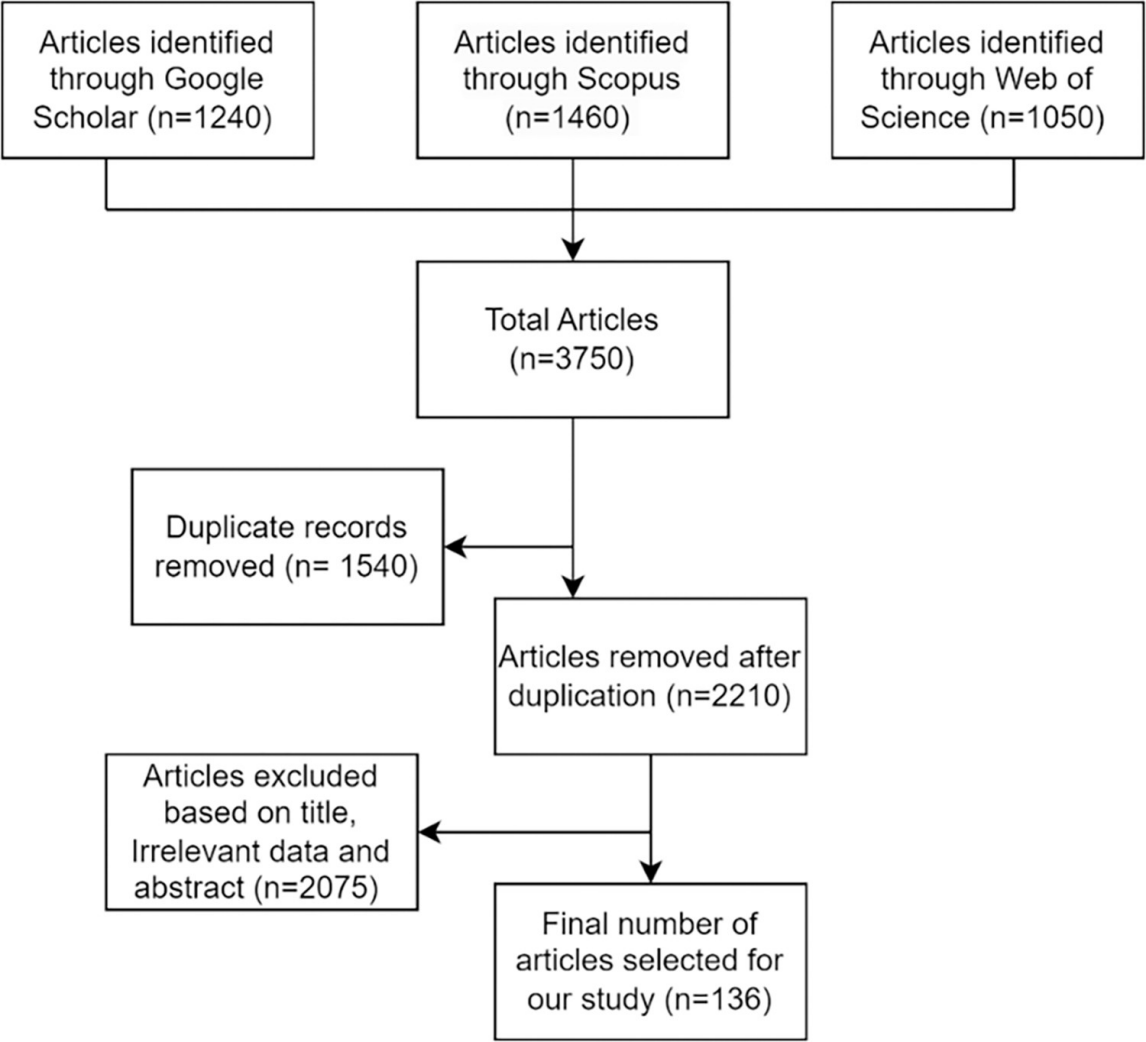
PRISMA Flowchart detailing the current 3-phase scoping review protocol and literature identification outcomes.

Overall, a total of 3750 articles from Google Scholar, Web of Science, and Scopus were identified (Fig 3). Following deduplication, this collection decreased to 2210 articles. Based on a thorough evaluation of article titles, abstracts and included data (from methodology section and Appendices), a further 2075 articles were excluded from further consideration. The final article cohort includes only articles published in English between January 1^st^ 2006 and May 31^st^ 2023, and independently adjudged (2 x groups of 2 authors) by the author team as being directly relevant to the topic (Table 2). Quality assessment of included research papers, while not strictly considered necessary for scoping reviews, is critical in assessing literature consistency, validity, and overall credibility [18]. Accordingly, the authors employed a non-summative 5-point quality system adapted from Wylde et al (2017). Our tool consisted of five items used to assess 1. relevance to scoping review question (based on full paper review), 2. selection bias (i.e., input data sources provided), 3. transferability (open-source data usage, open code), 4. bias due to missing data and/or lack of clarity, and 5. consideration of analytical confounding, model overfitting and/or study limitations. Each item was rated as adequate, inadequate or not reported, with only articles attributed as being “adequate” across all five criteria adjudged to be acceptable quality for narrative inclusion and data synthesis.

## 3. Review of identified relevant literature

### 3.1 Study characteristics

**Fig 4:**
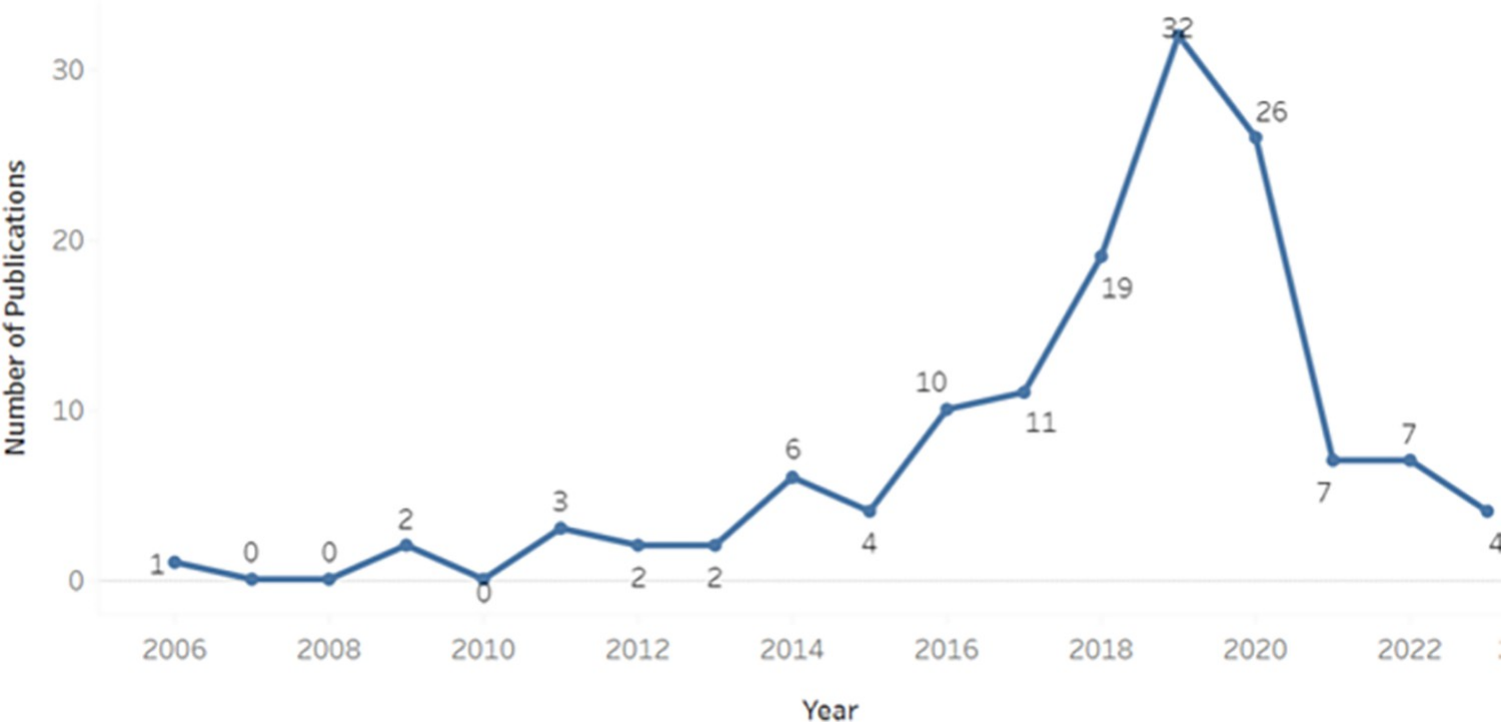
Timeline of identified article numbers per annum, January 2006 –May 2023.

Overall, 136 relevant studies were identified between January 2006 and May 2023, with the research timeline, based on article number per annum, presented in Fig 4. As shown, the annual number of publications remained relatively constant from 2006 to 2014, after which a marked increase was observed, reaching a peak in 2019 (n = 32). Subsequently, there was a significant decrease in publications after 2019, likely due to the COVID-19 pandemic and its impact on data accessibility, in addition to a shift in research focus among researchers in the realms of biomedical image analyses and classification algorithms.

**Fig 5:**
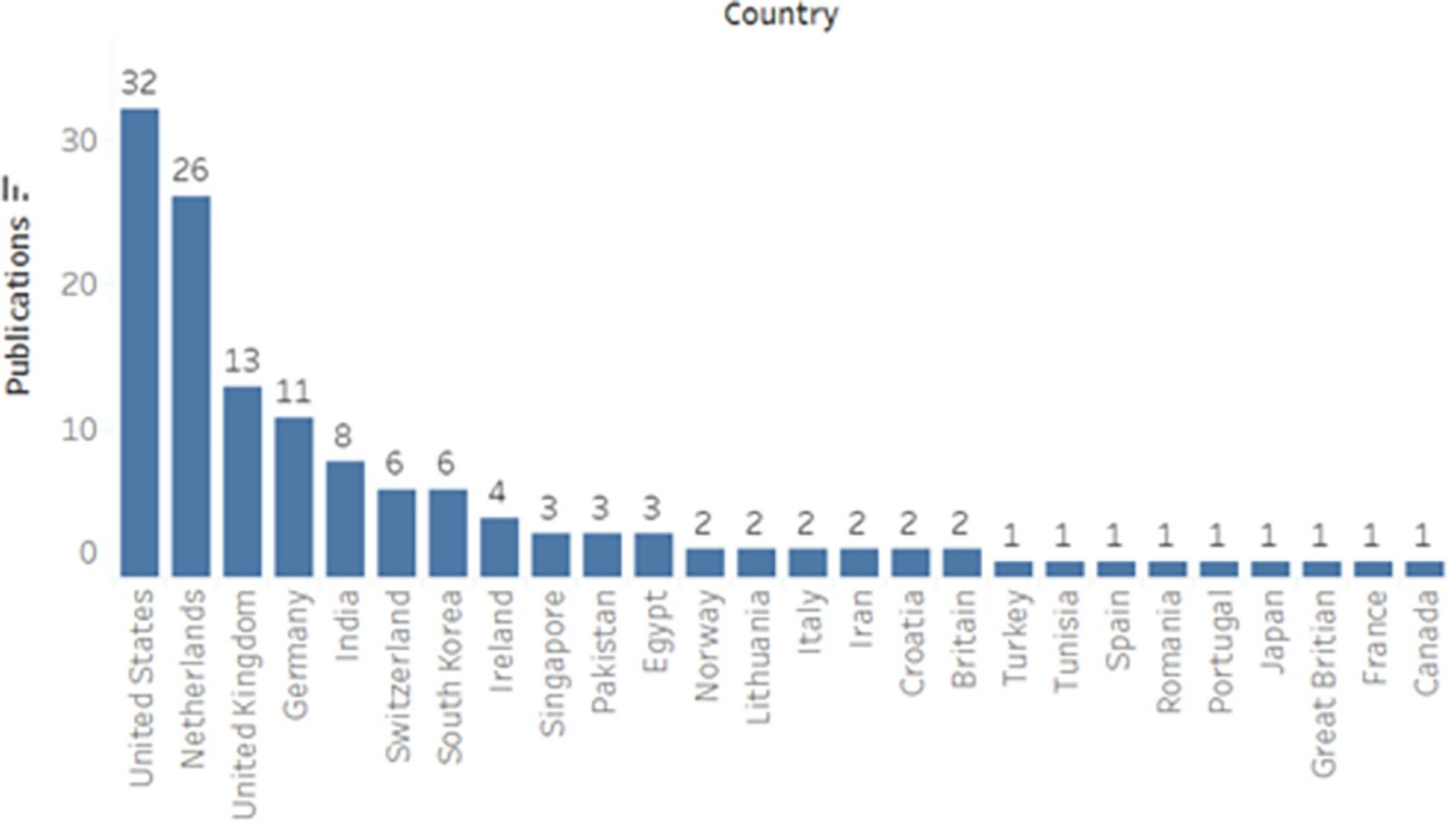
Identified articles delineated by country of origin (based on first and corresponding authors and origin of study dataset).

**Fig 6:**
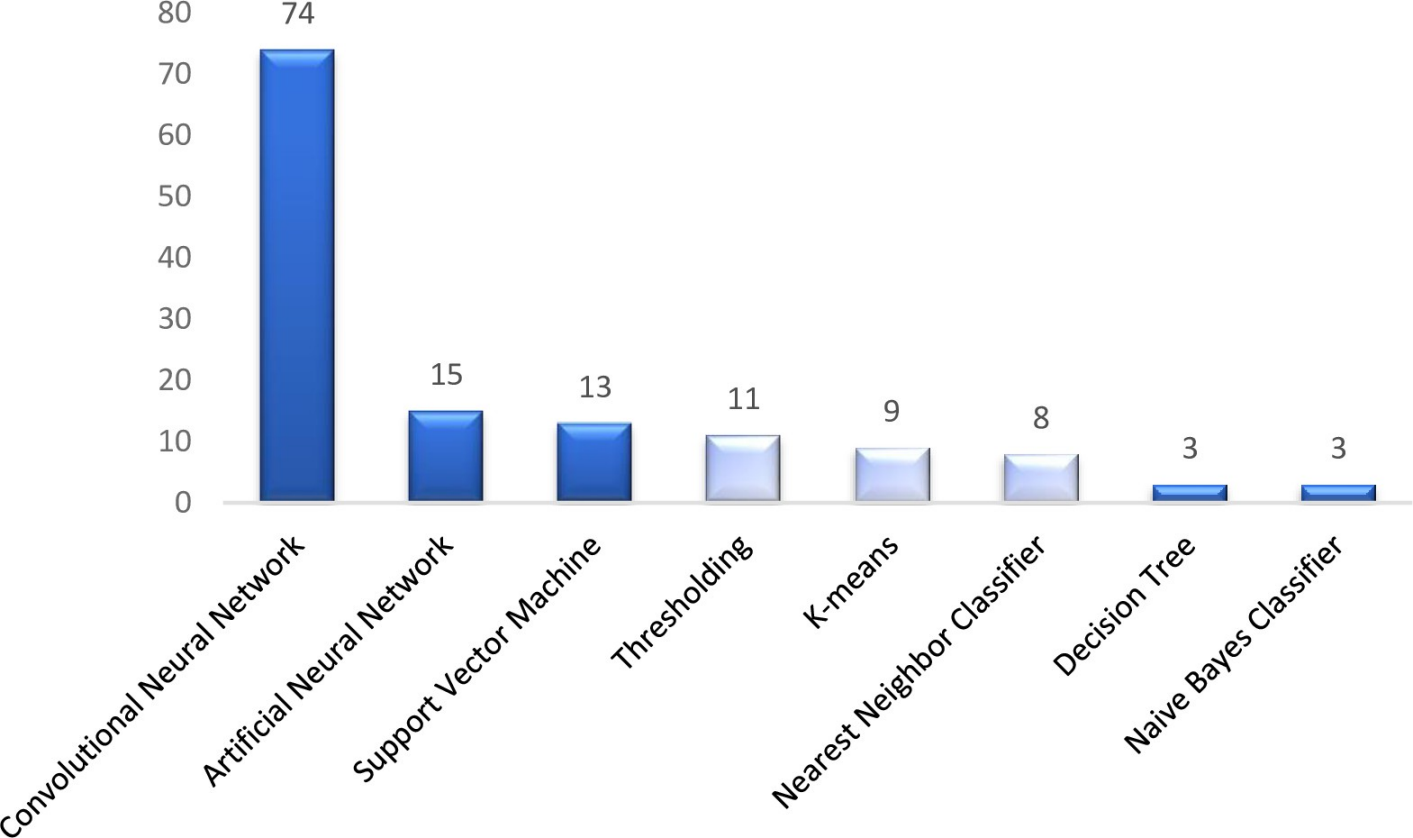
**Model architectures (dark blue) and methods (light blue) used for white blood cell classification.** (Note–Several comparative studies compared >1 method and/or developed ensemble architectures).

Overall, 26 countries were represented by identified studies, with the highest number of studies emanating from the United States (n = 32) and The Netherlands (n = 26) (Fig 5). High-income countries were, perhaps unsurprisingly, well represented, likely due to the availability of large datasets for training and testing, in addition to increasingly mature/well-funded national healthcare systems. As shown (Fig 6), 8 overarching model architectures and methods were employed for classification, including both traditional machine learning and deep learning models. Traditional machine learning models included Decision Trees (DT), K-means, Naive Bayes Classifier (NBC), Nearest Neighbor Classifier (NNC), Support Vector Machines (SVM), Artificial Neural Networks (ANN), and thresholding techniques. Within the deep learning domain, convolutional neural networks were the most frequently employed approach, likely due to their high performance and accuracy (compared and presented in Sections 3.2–3.6).

In total, 27 datasets were specifically referenced across the identified relevant studies ranging from 21 images [88] to 92,800 images [73] (Table 3), including ALL-IDB, one Private Dataset [60], CellaVision, AA-IDB2, Hayatabad Medical Dataset, Isfahan Al-Zahra and Omid hospital, ALL-IDB2/Leishman stained peripheral blood smears, one Public Dataset [73], BCCD, Kaggle, LISC and BCCD, Jiangxi Tecom Science Corporation/CellaVision/Bsisc/LISC, KMC hospital Manipal India, Hybrid-Leukocyte database/e Hybrid-Slide database, Acquired from Sixth People’s Hospital of Shenzhen, SMC-IDB/IUMS-IDB/ALL-IDB, and SBILab. The full list of datasets along with the corresponding dataset size (number of images) is provided in Table 3. Just two studies [6] specifically referred to the use of thin blood smear images, with the remaining studies either expressly referring to the use of thick blood smears or not reporting on smear type; this is notable, as thick features have inherent advantages over thin features in WBC classification outcomes.

**Table 3:** Identified white blood cell datasets used for classification studies 2006–2023.

Dataset	No of Images
ALL-IDB [57]	130
Private Dataset [60]	70
CellaVision [47]	100
AA-IDB2 [8]	108
Hayatabad Medical Dataset [63]	1030
Isfahan Al-Zahra and Omid hospital [64]	312
Private Dataset [66]	431
ALL-IDB2/ Leishman-stained peripheral blood smears [59]	160/160
CellaVision [31]	450
Public Dataset [73]	92,800
BCCD [41]	12,444
Kaggle [42]	12,444
BCCD [33]	12,500
BCCD [76]	375
Kaggle/LISC [77]	12,500/400
LISC and BCCD [78]	6250
Jiangxi Tecom Science Corporation/ CellaVision/ Bsisc/ LISC [79]	300/ 100/ 268/ 257
KMC hospital, Manipal, India [80]	280
ALL-IDB [85]	108
Hybrid-Leukocyte database/ e Hybrid-Slide database [86]	891/ 377
Acquired from Sixth People’s Hospital of Shenzhen [88]	21
Kaggle [89]	12,494
SMC-IDB/ IUMS-IDB/ ALL-IDB [92]	367/ 195/ 108
BCCD [94]	12447
SBILab [97]	76
BCCD [101]	2487
Kaggle [103]	12,444

### 3.2 White blood cell classification using conventional machine learning

Various studies have explored conventional machine learning methods for WBC classification, which for the purpose of clarity, the authors have organized into pre-processing-based techniques (Section 3.3.1), feature extraction (Section 3.3.2), and classification (Section 3.3.3). A total of 39 studies were identified, with 13 papers (33.3%) focused on pre-processing techniques, 15 papers (38.5%) delving into feature extraction methods, and 11 papers (28.2%) emphasizing classification techniques for WBC classification. This distribution of approaches and objectives is evident in Tables 4–7, highlighting diverse emphases on these sub-processes within the conventional machine learning domain for classifying WBCs.

**Table 4:** WBC nuclei detection accuracy, specificity and sensitivity in blood smear images (n = 10).

Author	Year	Method	Accuracy (%)	Specificity (%)	Sensitivity (%)
Safuan et al. [56]	2018	Otsu thresholding and watershed marker	98.87	96.87	99.1
Huang et al. [57]	2012	Otsu thresholding	98	-	-
Danyali et al. [58]	2015	Fuzzy divergence threshold	98		
Manik et al. [59]	2016	Adaptive thresholding	98.9		
Li et al. [60]	2016	Dual thresholding	97.85		
Wang et al. [61]	2106	Spectral and morphologic	90		
Negm et al. [62]	2018	K-mean clustering	99.15	99.52	99.34
Khosroseresliki et al. [45]	2017	Simple thresholding	93.7	-	-
Bouchet et al. [46]	2019	Fuzzy set algorithm	99.3	-	-
Jha et al. [47]	2019	Hybrid model based on Mutual Information	98.7	98	98

**Table 5:** Support Vector Machine accuracy, specificity and sensitivity for WBC classification (n = 4).

Author	Year	Method	Accuracy (%)	Sensitivity (%)	Specificity (%)
Duan et al. [8]	2019	Support Vector Machine	98.3	-	-
Sajjad et al. [6]	2016	Support Vector Machine	98.6	96.2	98.5
Amin et al. [63]	2015	K-means and SVM	97	84.3	97.3
Again et al. [64]	2018	Support Vector Machine	94	95.77	97.87

**Table 6:** Conventional machine learning approaches for WBC classification (n = 6).

Author	Year	Method	Accuracy (%)	Sensitivity (%)	Specificity (%)
Gautam et al. [20]	2016	Naïve Bayesian classifier	80.88	-	-
Tantikitti et al. [65]	2015	Decision Tree	92.2	-	
Rawat et al. [66]	2017	PCA-SVM	94.6	97	88
Shaikhina et al. [67]	2019	Decision Tree and RF	85	81.8	88.9
Abdeldaim et al. [68]	2018	K-NN	98.6	-	-
Mathur et al. [69]	2013	Naïve Bayesian Classifier	92.72	90	-

**Table 7:** Artificial Neural Network (ANN) accuracy, specificity and sensitivity for WBC classification (n = 7).

Author	Year	Method	Accuracy (%)	Sensitivity (%)	Specificity (%)
Hegde et al. [70]	2019	ANN	99	99.4	99.18
Manik et al. [59]	2016	ANN	98.9	-	-
Su MC et al. [71]	2014	ANN	99.11	97.3	98.2
Lee et al. [31]	2014	Hybrid Neural Network based Classifier	91	-	-
Rawat et al. [36]	2018	K-Means, ensemble artificial neural network (EANN)	95	-	-
Nazlibilek et al. [37]	2014	Neural Network Classifier	95	-	-
Sadeghian et al. [72]	2009	ANN	78	-	-

#### 3.2.1 Pre-processing-based ML techniques

Pre-processing-based techniques include methods that manipulate and enhance raw data prior to further analysis. In the context of WBC classification, these techniques play a critical role in refining images to enable accurate categorization. Rosyadi et al. [19] used optical microscopy to generate blood samples images, with their method comprising four stages: image pre-processing, segmentation, feature extraction, and classification. In the first phase of image pre-processing, images were transformed from RGB to grayscale and binary images. Subsequently, in the second phase, resizing, cropping, and edge detection were applied to all images. Five geometrical features were considered in the feature extraction phase that represent important geometric characteristics of the segmented cells: normalized area, solidity, eccentricity, circularity, and normalized perimeter. These characteristics help differentiate various types of WBCs and enable accurate classification through K-means clustering. The study focus was analysis of each feature for accuracy. After experimentation, it was concluded that the circularity feature was most significant as it achieved the highest accuracy (67%), with the eccentricity feature having the lowest accuracy of 43%.

Gautam et al. [20] also presented a technique initiated via pre-processing of microscopic images. Pre-processing involved conversion of RGB (Red, Green, Blue) images to grayscale, contrast stretching, and histogram equalization. Subsequently, they applied segmentation through Otsu’s thresholding method, followed by geometrical feature extraction, including perimeter, area, eccentricity, and circularity. Finally, a Naïve Bayes classifier was used for classification with the maximum likelihood method, achieving 80.88% accuracy. Savkare et al. [21] presented an alternative method for blood cell segmentation; their pre-processing approach employed median and Laplacian filters to enhance image quality. After pre-processing, images were transformed from RGB to HSV (Hue, Saturation, Value) color space. Subsequently, K-mean clustering was applied for segmentation of blood cells. Furthermore, they used morphological operation and a watershed algorithm to refine cell separation. The proposed method through K-mean clustering acquired an accuracy of 95.5%.

#### 3.2.2 Feature extraction-based ML techniques

Typically, a differential counting method of WBCs is used to assess a patient’s immune system. This method involves using flow cytometry and fluorescent markers, which may disturb the cell due to repetitive sample preparation. Accordingly, label-free techniques that use imaging flow cytometry and ML algorithms to classify unstained WBCs are considered a more effective approach. Toh et al. [22] previously reported a mean F1-score of 97% across B and T subtypes, with each individual subtype achieving a distinct F1 score of 78%. Tsai et al. [23] proposed a multi-class support vector machine (SVM) approach to hierarchically identify and categorize blood cell images; segmentation was implemented on digital images to retrieve geometric features from each segment, enabling identification and classification of different blood cell types. The experimental outcomes were compared with manual results, revealing that the proposed method significantly outperformed manual classification with an accuracy of 95.3%. Likewise, Şengür et al. [24] presented a model combining image processing (IP) and ML techniques for WBC classification. Shape-based features and deep features were utilized to describe WBCs, with a long-short-term memory (LSTM) model applied to a dataset comprising 349 blood smear images with 10-fold cross-validation, from which 35 geometric and statistical features were extracted. More recently, Elen and Turan [25] compared six ML techniques (decision tree classifier, Random Forest, K-Nearest Neighbor, Multinomial Logistic Regression, Naïve Bayes, and SVM) for WBC categorization. Using shape-based features, an accuracy of 80% was achieved, while deep features achieved 82.9% accuracy. Overall, Multinomial Logistic Regression returned the highest precision rate of 95%, followed by Random Forest.

Huang et al. [26] presented a technique for WBC segmentation, delineating their approach into three phases: nucleus segmentation and recognition, feature extraction, and classification. A leukocyte (WBC) nucleus enhancer (LNE) was used to enhance the contrast of nucleus colors for segmentation, after which, multiple levels of Otsu’s thresholding were applied, effectively preserving only the WBCs and suppressing other cell types. During the feature extraction phase, a gray-level co-occurrence matrix was employed from which 80 texture features were extracted. Subsequently, they incorporated shape-based features, including compactness and roughness, after which Principal Component Analysis (PCA) was used to reduce feature dimensions. Classification was achieved using a genetic-based parameter selector (GBPS) with 50X cross-validation, resulting in 95% classification accuracy. Yampri et al. [27] also segmented out the WBCs via automatic thresholding (i.e., segregation of cell nucleus from cytoplasm) and feature extraction. Eigen cells were used to remove segments by applying the following approach: conversion of cell image to vector, computation of mean and covariance of vector, computation of eigen values and eigen vectors. Principle component analysis (PCA) was used to transform high dimensional eigen space to significantly lower dimensional space, with 92% classification accuracy achieved.

#### 3.2.3 Classification-based (focused) ML techniques

Tavakoli et al. [28] developed a three-phase ML method for improved WBC classification delineated as follows: nuclei/cytoplasm detection, extract features, and classification. A novel process was designed to segment the entire nucleus, while cytoplasm segmentation involves location detection inside the convex region. In the next phase, four unique colors and three shape features were extracted, and finally, in the last phase, SVM was used for WBC classification. Overall, 94.2% accuracy was achieved on the BCCD dataset, 92.2% with LISC dataset, and 94.65% with the Raabin-WBC datasets, however, hyperparameter issues were encountered.

An innovative "Computer-aided diagnostic system" method was proposed by Malkawu et al in 2020 with this process utilizing a hybrid approach, whereby CNN was employed as a feature extractor. The performance of several classifiers was measured, with Random Forest (RF) outperforming other classifiers based on a 98.7% accuracy [29]. A similar multi-approach (i.e., comparison of several ML algorithms) by Gupta et al. [30] presents an optimized form of the Binary Bat algorithm inspired by bat echolocation techniques. Using OBBA (Table 3), dimensionality reduction was achieved by eliminating ≥11 similar features. Four classifiers (KNN, Logistic Regression, RF, and DT) were applied for WBC classification, demonstrating highest performance, with a mean accuracy of 97.3%, thereby surpassing other optimizers like the Optimized Crow Search Algorithm (OCSA), which attained an accuracy of 92.8% and the Optimized Cuttlefish Algorithm (OCFA), with an accuracy of 95.2%.

Lee [31] proposed an innovative approach to image segmentation based on grey-level thresholding, based on previous findings that cell-type specific reaction of the cells produces adequate evidence to allow precise classification. This method was tested on a dataset comprising 1149 WBCs from 13 altered, clinically significant categories. Cells were randomly selected from 20 blood smear images obtained from leukemia patients, with cell sorting based on quantitative volumes in the segmented images producing a classification accuracy of 82.6%.

#### 3.3 White blood cell classification using deep learning techniques

Wibawa et al. [32] proposed a DL model for classifying two WBC types, comparing the results with conventional machine learning methods (support vector machines), using nine features for classification. The authors report that deep learning significantly exceeded conventional ML methods, achieving highest accuracy of 95.5%. Toğaçar [33] introduced a WBC classification approach based on the coefficient and ridge feature selection method utilizing a CNN model with GoogleNet and ResNet50 for feature extraction. They achieved 97.95% accuracy for WBC classification and counting. Likewise, CNN was employed to identify and classify segmented WBC images as being “granular” or “non-granular”. Subsequently, granular cells were further categorized into eosinophils and neutrophils, while non-granular cells were classified as lymphocytes and monocytes [34]. To enhance dataset robustness, augmentation approaches were implemented, resulting in improved accuracy for both binary and multi-classification of blood cell subtypes, leading to 98.51% precision for binary WBC classification and 97.7% precision for subtype classification.

Lippeveld et al. [35] employed a relatively small dataset to examine human blood samples using image flow cytometry, with two models used to identify eight WBC types and eosinophils exclusively. ML models were applied to both datasets to classify human blood cells with 5-fold cross validation. Random Forest (RF) and Gradient Boosting (GB) were used for the first model, while deep learning CNN architecture (ResNet and DeepFlow (DF)) were employed for the second model. On the WBC dataset, results demonstrated a relatively balanced accuracy of 77.8% and 70%, while similarly for the eosinophil dataset, a balanced accuracy of 87.1% and 85.6% was achieved. DF outperformed the RN architecture on the WBC dataset, acquiring a classification accuracy of 70.3% compared to RN’s 64.9%.

Rawat et al. [36] introduced another deep learning method employing the DenseNet121 model for classification of several WBC types—The proposed model was estimated, with an accuracy of 98.84%. Results indicate that the DenseNet121 model with a batch size of 8 exhibited the highest overall performance. The dataset, consisting of 12,444 images, was obtained from Kaggle. Nazlibilek et al. [37] proposed a DL-based method that leveraged image variation operations and generative adversarial networks (GAN) for accurately classifying WBCs into five distinct types. Likewise, Sadeghian et al. [38] developed a two-stage model comprising an initial alteration using a pre-trained model, followed by the integration of a ML classifier. They employed the BCCD dataset, a downscaled blood cell detection dataset, and achieved a precision of 97.03%. Likewise, Sadeghian et al. [38] developed a two-stage model comprising an initial alteration using a pre-trained model, followed by the integration of a ML classifier. They reported 97.03% classification accuracy on the BCCD dataset, a downscaled blood cell detection dataset. Macawile et al. [39], utilized Convolutional Neural Networks (CNNs) to effectively classify and count WBCs in microscopic blood images. Among the proposed models AlexNet, GoogleNet, and ResNet-101. AlexNet performed better than the other two. It demonstrated an overall accuracy of 96.63%, albeit with a relatively lower sensitivity rate of 89.18%.

Liang et al. [40] introduced an innovative approach that merges convolutional neural networks (CNNs) with recurrent neural networks (RNNs). This fusion, termed the CNN-RNN framework, enhances understanding of image content and structured feature learning, enabling end-to-end training for comprehensive medical image data analysis. They applied transfer learning, adapting pre-trained weight parameters from the ImageNet dataset for the CNN segment. Additionally, a customized loss function was integrated to expedite training and achieve precise weight parameter convergence. Experimental results indicate a classification accuracy of 90.79%. More recently, Sharma et al. [41] presented yet another CNN-based classification methodology, achieving an impressive 96% accuracy for binary classification and 87% accuracy for multiclass classification.

Togacar et al. [42] employed a very different DL approach to WBC classification by using a computer-aided automated approach. Utilizing Regionally Based Helixal Neural Networks, their study effectively classified and differentiated WBCs, achieving an objectively high level of classification accuracy (99.52%). Toğaçar et al. [33] also introduced a method composed of three essential phases. In the initial stage, CNN models specifically AlexNet, GoogleNet, and ResNet-50 are utilized as feature extractors. Subsequently, the features extracted from these CNN model layers are fused. In the second phase, the technique incorporates feature selection methods, including MIC and Ridge Regression. In the third phase, these selected features are amalgamated. The overlapping features derived from the MIC and Ridge Regression techniques are then classified using the QDA method. This integrated approach achieves a remarkable overall success rate of 97.95% in classifying WBCs.

Mohamed [43] introduced an alternative method for the identification and classification of blood cells based on CNN. The study presented two distinct approaches for classifying WBCs. In the initial approach, CNN was employed with transfer learning, utilizing pre-trained weight parameters applied to the images. In contrast, the second approach utilized Support Vector Machines (SVM) for the classification process. The classification results demonstrated a remarkable 98.4% accuracy for CNN and 90.6% accuracy for SVM. The classification results of CNN are higher compared to SVM. Yao et al. [44] introduced a CNN-based approach for the classification of WBCs. In their method, CNN integrated an optimizer to adaptively adjust parameters such as the learning rate, leveraging the efficient net architecture. The utilization of the optimizer responded to changes in loss and accuracy. Their proposed model demonstrated exceptional performance, achieving an impressive accuracy of 90%.

Khosrosereshki et al. [45] developed an R-CNN-based model to identify neutrophils, eosinophils, monocytes, and lymphocytes, with two models employed, namely Faster RCNN and Yolov4. Faster RCNN obtained an accuracy of 96.25%, while Yolov4 was slightly lower at 95%. Likewise, Bouchet et al. [46] utilized the Inception Recurrent Residual Convolutional Neural Network (IRRCNN) model, an advanced hybrid architecture based on residual networks and RCNN principles. The proposed IRRCNN demonstrated exceptional accuracy in experiments, achieving a 100% accuracy rate for WBC classification.

Jha et al. [47] developed a leukemia detection module specifically designed for blood smear images with their multi-phase detection process comprising pre-processing, segmentation, feature extraction, and classification. The segmentation step utilizes a hybrid model based on Mutual Information (MI), which combines results from the active contour model and fuzzy C means algorithm. Subsequently, statistical and Local Directional Pattern (LDP) features are extracted from the segmented images. These features are then fed into a novel Deep CNN classifier based on the proposed Chronological Sine Cosine Algorithm (SCA) for classification purposes. Testing used blood smear images from the AA-IDB2 database, with simulation results indicating that the developed classifier achieved an accuracy of 98.7%.

Ullah et al. [48] introduced a 3D-CNN feature-based CBVR system that is highly efficient and effective for retrieving similar content from vast video data repositories. After an in-depth exploration of its effectiveness in representing sequential frames, they selected middle layer features of a 3D-CNN model. Leveraging a mechanism for selecting convolutional features, only the active feature maps from the CNN layer that correspond to the ongoing event in the frame sequence are chosen. To condense the size of the extracted high-dimensional features for streamlined retrieval and expedited storage, they introduced the concept of hashing. These high-dimensional features are represented in compact binary codes through PCA, ensuring efficient search and reduced storage requirements for WBCs classification. For the classification of WBCs, the achieved accuracy is 85%.

Imran et al. [49] conducted a study involving the utilization of a four-hidden-layer feed-forward DNN and CNN. The research also extensively examines the impact of Mel-Frequency Cepstral Coefficients (MFCC) and Filter Bank Energies (FBE)features trained with various context sizes on two deep learning models, evaluated under normal, slow, and fast speaking rates. Micro-level analysis of results was conducted, revealing that the four-hidden-layer CNN slightly outperforms the DNN in classifying WBCs. The CNN achieved an accuracy of 83% in classifying WBCs. Kastrati et al. [50] introduced a convolutional neural network with three hidden layers, each having 1024 neurons, showcasing excellent performance in white blood cell classification on the INFUSE dataset, achieving accuracy of 78.10%.

Ullah et al. [51] introduced an innovative conflux Long Short-Term Memory (LSTM) network for WBC classification. The framework involves four stages: 1) frame-level feature extraction, 2) feature propagation through the conflux LSTM network 3) pattern acquisition and correlation computation, and 4) action classification. The process begins with extracting deep features using a pre-trained VGG19 CNN model from frame sequences for each view. Extracted features then undergo conflux LSTM processing to learn unique view-specific patterns. Interview correlations are computed by utilizing pairwise dot products from LSTM outputs across views, thus acquiring interdependent patterns. The VGG19 CNN model achieved a classification accuracy of 88.9%. Meanwhile, Banik et al. [52] recently presented a CNN)-based WBC image classifier which merges features from both the initial and final convolutional layers, while utilizing input image propagation through a convolutional layer to enhance performance. A dropout layer is added to counter overfitting, resulting in a classification accuracy of 98.61%. Another CNN-based approach has been developed by Ku et al. [53] who propose an automated system for leucocyte classification using a dual-stage CNN. A dataset of 2,174 patch images was collected for training and testing purposes, with the dual-stage CNN used to classify images into 4 classes, achieving an overall accuracy of 97.06%.

Karthikeyan et al. [54] introduced the Leishman-stained function deep classification (LSM-TIDC) model for WBC classification. Interestingly, the LSM-TIDC method explores the potential of interpolation and Leishman-stained function without the need for explicit segmentation, which if successfully implemented, effectively eliminates false regions in multiple input images. Following image pre-processing, relevant features are extracted through multi-directional feature extraction, with a system then developed, utilizing a transformation invariant model to extract nuclei and subsequently employing convolutional and pooling characteristics for cell classification. Method testing was conducted on the Kaggle dataset, and classification accuracy of 94.42% was achieved.

Acevedo et al. [55] used a large dataset of 17,092 peripheral blood cell images across eight classes gathered using the CellaVision DM96 analyzer. Pathologist-verified ground truth data were used to train two CNN architectures: Vgg-16 and Inceptionv3. In the first setup, networks acted as feature extractors for an SVM, achieving test accuracies of 86% (Vgg-16) and 90% (Inceptionv3). In the second setup, fine-tuning resulted in “end-to-end” models, yielding 96% accuracy (Vgg-16) and 95% accuracy (Inceptionv3).

Upon comparing the identified 136 relevant studies, as a general observation, detection of WBCs through conventional methods (ML) tends to focus on cell segmentation after data pre-processing, with segmented data then typically employed for feature extraction in WBC classification. Accordingly, the traditional ML methods were associated with better results as accurate identification of WBCs is impractical in the absence of efficient segmentation, thus resulting in higher levels of classification accuracy (Tables 4–8). Research teams employed a range of methods for data segmentation and obtained a range of classification accuracies; while some conventional models achieved up to 99% accuracy, accuracy was shown to decrease in concurrence with decreasing dataset size (e.g., Lippeveld et al. [35]). Deep learning models exhibited improved performance for more extensive datasets and exhibited higher levels of accuracy in concurrence with increasingly large datasets (Table 6). Several authors implemented a combination of different datasets, to probe the accuracy of their models on unknown datasets (i.e., blind testing). Deep learning models have represented a significant breakthrough in myriad domains and as shown in the identified literature, the use of traditional machine learning models within biomedical applications in general, and WBC classification in particular is undoubtedly shifting toward the use of deep learning models based on dataset size. However, deep learning algorithms (and associated research) are now in a significantly more advanced phase, with proven capacity to solve increasingly complex problems with higher performance. Notwithstanding, there is a clear gap in the use of the latest advances in deep learning, including the use of transfer knowledge and meta-learning processes.

**Table 8:** Deep learning model accuracy, specificity and sensitivity for WBC classification (n = 36).

Author	Year	Method	Accuracy (%)	Sensitivity (%)	Specificity (%)
Macawile et al. [39]	2018	AlexNet	96.63	98.85	99.61
Liang et al. [73]	2018	CNN + RNN	91	-	-
Sharma et al. [41]	2019	CNN	97	94	98
Togacar et al. [42]	2019	CNN	97.78	-	-
Mohamed et al. [74]	2020	Pre-trained Deep Learning Models	97.03	71	91
Ergen et al. [33]	2020	CNN, Feature Selection	97.95	98	97.75
Zhao et al. [75]	2021	TWO-DCNN	96	-	-
Cinar et al. [76]	2021	Alexnet- GoogleNet-SVM	99.73, 98.23	98.75	-
Wang et al. [77]	2019	CNN Architecture SSD and YOLOv3	90.09	-	-
Kutlu et al. [14]	2020	R-CNN	97.52	88.9	-
Fan et al. [78]	2019	ResNet50	98	-	-
Hegde et al. [79]	2019	Pre-trained AlexNet model	98.9	98.6	98.7
Acevedo et al. [80]	2019	Pre trained CNN	96.2	-	-
Qin et al. [81]	2018	Deep Residual Learning	76.84	-	-
Tiwari et al. [82]	2018	Double CNN model	97	83	-
Hung et al. [83]	2017	AlexNet and Fast R CNN Model	72	-	-
Naz et al. [84]	2017	CNN, faster R CNN	94.71	95.42	99.27
Vogado et al. [85]	2018	CNN with SVM	99.20	99.2	-
Habibzadeh et al. [86]	2018	ResNet and Inception	99.46	99.89	-
Song et al. [87]	2014	CNN	94.5	87.26	-
Fatih et al. [88]	2019	MRMR feature selection -ELM and CNN	97.37	-	-
Rehman et al. [89]	2018	Deep CNN	97.78	-	-
Bani-Hani et al. [90]	2018	CNN with the optimized genetic method	91	91	97
Di Ruberto et al. [91]	2020	Pre trained AlexNet	97.93	99.6	-
Loey et al. [92]	2020	Pre trained CNN AlexNet	100	100	98.2
Ma et al. [93]	2020	Generative Adversarial Network and residual neural network	91.7	92	-
Baydilli et al. [94]	2020	Capsule Networks	96.86	92.5	98.6
Tobias et al. [95]	2020	Faster Residual Neural Network	83.25	-	-
Kassani et al. [96]	2019	Hybrid DL based model	96.17	95.17	98.58
Baghel et al. [97]	2022	CNN	98.51	98.4	-
Shahzad et al. [98]	2022	CNN	98.44	99.96	99.98
C. Cheuque et al. [99]	2022	Multilevel CNN	98.4	98.3	-
Hosseini et al. [100]	2022	Convolutional Neural Network	97	94	98
Ramya et al. [101]	2022	CNN-PSO	99.2	94.56	98.78
Khalil et al. [102]	2022	CNN	98	-	-
Sharma et al. [103]	2022	DenseNet121	98.84	98.85	99.61

Comparative analysis of deep learning models applied to various large datasets revealed remarkably high levels of achieved accuracy across various studies (Table 9). Baghel et al. [97] demonstrated a high level of efficacy associated with the use of CNNs, achieving an accuracy of 98.51%, while Riaz et al. [117] used a Convolutional Generative Adversarial Network (GAN) to obtain a classification accuracy of 99.9% on the Catholic University of Korea dataset. Mosabbir et al. [118] addressed the challenging National Institutes of Health (NIH) dataset using CNN, attaining an accuracy of 97.92%. Tusneem et al. [119] also used CNN and demonstrated its strength, with a 99.7% classification accuracy. Kakumani et al. [120] utilized a pre-trained InceptionV3 model on the Kaggle dataset and achieved 99.76% classification accuracy.

**Table 9:** Comparative analysis of best performing (based on reported accuracy) deep learning models (n = 8).

Author	Year	Dataset	No of Images	Method	Accuracy (%)
Baghel et al. [97]	2022	Private dataset	-	CNN	98.51
Cheuque et al. [99]	2022	Private dataset	-	Multilevel CNN	98.4
Ramya et al. [101]	2022	Merged LISC and BCCD	-	CNN-PSO	99.2
Sharma et al. [103]	2022	Kaggle	12,444	DenseNet121	98.8
Riaz et al. [117]	2023	Catholic University of Korea /Public dataset	5000	deep convolutional generative adversarial network	99.9
Mosabbir et al. [118]	2023	National Institutes of Health (NIH) dataset	27,558	CNN	97.9
Tusneem et al. [119]	2023	AML Cytomorphology LMU	18,365	CNN	99.7
Kakumani et al. [120]	2023	Kaggle	12,515	Pre-trained inception v3	99.7

## 4. Limitations of previous studies and future challenges

ML/DL researchers have made significant advances in increasingly accurate classification of WBCs in recent years. Among all techniques based on SVM, Sajjad et al. [6] achieved maximum accuracy, sensitivity, and specificity of 98.6%, 96.2%, and 98.5%. Using KNN, Abdeldaim et al. [68] achieved maximum accuracy of 98.6%. Similarly, using ANN, Hegde et al. [70] acquired accuracy, sensitivity, and specificity of 99%, 99.4%, and 99.18%, respectively. Using DL methods, Loey et al. achieved maximum precision and sensitivity of 100% each and specificity of 98.2%; However, while many researchers achieved close to maximum performance, several limitations and constraints have been associated with previous and current techniques. Accordingly, the research community faces several fundamental obstacles in the field of MIA that must be accepted and resolved. These include the lack of easily accessible, large, high-quality datasets, a shortage of dedicated medical professionals, and the complexity of Transfer Learning and Deep Learning methods. Several DML strategies, mathematical and theoretical foundations are also a source of several challenges [96, 104], with unsupervised or semi-supervised systems needed to address these issues [105]. Moreover, TML and DL-based MIA applications and systems still have significant work to adopt “real-time application”.

### 4.1 Lack of publicly accessible datasets

The lack of publicly accessible datasets represents the primary issue affecting medical image analysis. Scientists need to inspire health organizations to address this problem, it would be beneficial if high-quality data were available to researchers. Initiatives promoting open data availability from various health organizations worldwide should also be encouraged. However, authorization should also be required (e.g., hospital data and conditional access to datasets). When data are readily available in large quantities, just like in other fields such as environmental science, weather forecasting, and bioinformatics, the issue becomes more relevant for research (e.g., video summarization [106], IoT [107], energy management [108], and so on). Acquiring very large, high-quality datasets with accurate labeling is crucial for MIA applications.

### 4.2 Generalization skills for trained predictors

Another very significant challenge associated with MIA and WBC identification and classification is the availability of appropriately trained predictors. A perfect learning method that balances computational efficiency with generalization capacity is required to solve this issue. To build a model with impressive generalization capabilities, a learning approach that incorporates true or random labels is necessary. This approach provides efficient training algorithms and practical tools to handle available datasets using accurate or arbitrary labels. Many MIA tasks, including identifying brain tumors, lung cancer, breast cancer, and leucocytes, have shown significant empirical success. Despite the inherent challenges posed by non-convex optimization, basic techniques such as stochastic gradient descent (SGD) can efficiently discover viable solutions, effectively minimizing training errors. More interestingly, the networks created in this manner have strong generalization capabilities [109], even when there are far more parameters than training data [110]. Only reducing the training error during model training is insufficient. The choice of global minima greatly impacts the generalization behavior of the predictor. It is crucial to select the appropriate algorithm to minimize training errors for better results. Different initialization, update, learning rate, and halting conditions for optimization algorithms will result in global minima with various degrees of generalizability.

### 4.3 Reliable methods for real-world scenarios

TML and DL approaches provide reliability to real-world health diagnosis systems [111]. However, MIA and leukocyte classification models requires expertise and technical skill. In the future, researchers should prioritize crafting accurate and trustworthy procedures applicable in real-world healthcare situations, eliminating the necessity for medical specialists. Real-world health diagnosis systems greatly gain from the dependability of Machine Learning (ML) and Deep Learning (DL) approaches [111]. Yet, constructing exact models for Medical Image Analysis (MIA) and leukocyte classification necessitates a high degree of expertise and technical proficiency. As research advances, it becomes crucial for researchers to tackle the task of developing reliable procedures that can smoothly integrate into real-world healthcare environments, reducing the reliance on specialized medical professionals. This involves tackling issues related to model generalization, data variability, interpretability, and ensuring consistent performance across diverse patient populations and clinical scenarios.

## 5. Future research directions

The biomedical engineering and research community should dedicate substantial effort to support MIA, particularly leukocyte examination in blood images, due to the significant challenges faced by the MIA community, as detailed in section V.

**i**. **Data augmentation methods to complete the dataset deficit**.

This work addresses the issue of limited dataset availability in MIA and leucocyte classification. We present data augmentation approach and leverage transfer learning algorithms to enhance the identification of WBCs.

**ii**. **Technical skills and medical experience required**.

TML and DL models have shown significant potential for computer-aided MIA-based diagnostic applications, and popular open-source frameworks like TensorFlow, Caffe, and Keras offer access to these advanced models [121]. Developing effective machine learning models for medical image analysis (MIA) requires careful consideration and expertise in the clinical and medical domains. It is essential to choose and train the suitable model to achieve accurate and reliable results in MIA applications.

**iii**. **Resource-aware DL models for classifying leukocytes**.

Medical Image Analysis (MIA) with the adoption of advanced DL models like GANs, R-CNN, Fast R-CNN, and faster R-CNN, along with the integration of TML and DL methods. These models have shown superior performance in tasks like brain tumor detection, leukocyte classification, breast cancer diagnosis, and various other MIA applications. However, their biggest concerns are the significant memory needs and computational costs. Therefore, it is necessary to investigate the computationally and environmentally friendly TML and DL models for leukocyte analysis in blood images.

**iv**. **Models for the detection and classification of leukocytes**

DNNs provide a superior alternative to conventional learning techniques. The end-to-end models, especially CNNs, stem from their efficient process and the capability to classify leucocytes into five classes. These models compete with complex MIA models built on DNN based on data-driven learning methodologies. WBC detection and categorization in images can also be accomplished using a variety of end-to-end designs [122–124].

**v**. **TML AND DL universal evaluation in MIA**

The MIA research community often relies on subjective evaluation methods, which can be challenging, inefficient, and prone to errors. Therefore, comprehensive evaluation techniques that can automatically assess the effectiveness of Traditional Machine Learning (TML) and Deep Learning (DL) models for MIA from various views.

**vi**. **Vision Transformers and Vision Formation Models**

While Vision Transformers (ViTs) were not included in the current review, they represent a likely cutting-edge approach for the future of white blood cell (WBC) classification (and other forms of imagery analyses), employing an advanced self-attention mechanism to extract crucial features from input images. Additionally, ViTs leverage transfer learning by incorporating pre-trained model weights, further boosting their performance. This dual approach meticulously captures subtle features, significantly enhancing the precision and accuracy of WBC classification—a major advancement in the realm of medical imaging. Likewise, vision foundation models are powerful generative deep learning models trained on large datasets for classification, segmentation, and detection, and will likely become a frequently employed approach for medical imaging in future.

## 6. Conclusion

We provide a comprehensive review of the TML and DL techniques applied to WBCs classification. We thoroughly explored and compared various methods for WBC categorization in this context. The data for this research is compiled from 136 primary papers published between 2006 and 2023. These papers encompass TML and DL methodologies for leukocyte classification and their applications in medical diagnosis. The comprehensive analysis of these studies reveals the significant contributions of TML and DL techniques to MIA. The main objective of this work is to identify and synthesize the myriad TML and DL applications in MIA, particularly in the domain of leucocyte classification in blood smear images. This research aims to provide valuable insights into the complex characteristics of TML and DL in MIA by thoroughly analyzing existing literature. Based on literature review outcomes, Deep Learning models like CNNs for image classification and GANs for data augmentation should be increasingly employed to negate the limitations (e.g., time) and human biases/inaccuracies associated with manual classification used. The study’s results emphasize the importance of conducting more research on using TML and DL methods effectively in MIA and classifying leucocytes in blood smear images. Besides leucocyte classification, this study explored applications for advanced DL models. Collecting all these data in this study will help the research industry by indicating where they should focus their future investigation of TML and DL models for MIA. These methods have the potential to lead to significant advancements in speech analysis, natural language processing (NLP), and medical imaging in the future. In addition to WBCs, TML and DL approaches are employed to identify and categorize various MIA domains, such as the analysis of MRI, CT, X-ray, and ultrasound images. Blood smear images are a growing field in MIA that has drawn attention from the research community over the past three decades. Additionally, we recognized the problems, instructions, and solutions for the developments of TML and DL models in MIA, notably for classifying WBCs in blood smear images. The potential of TML and DL approaches will be used to expand our research to include different MIA domains, including MRI, CT, Ultrasound, and X-ray images.

## Supporting information

S1 Checklist PRISMA 2020 flow diagram for new systematic reviews which included searches of databases and registers only. (DOCX)

S2 Checklist Preferred Reporting Items for Systematic reviews and Meta-Analyses extension for Scoping Reviews (PRISMA-ScR) checklist. (DOCX)

S1 Dataset (XLSX)

## References

[1] Pandey P., Pallavi S., and Pandey S. C., ‘‘Pragmatic medical image analysis and deep learning: An emerging trend,” in Advancement of Machine Intelligence in Interactive Medical Image Analysis. Singapore: Springer, Jan. 2020, pp. 1–18. [Online]. Available: http://www.springer.com/series/16171, doi: 10.1007/978-981-15-1100-4_1

[2] Asgari Taghanaki S., Abhishek K., Cohen J. P., Cohen-Adad J., and Hamarneh G., ‘‘Deep semantic segmentation of natural and medical images: A review,” Artif. Intell. Rev., vol. 4, pp. 1–42, Jun. 2020.

[3] Rahman M., Antani S., and Thoma G., ‘‘A learning-based similarity fusion and filtering approach for biomedical image retrieval using SVM classification and relevance feedback,” IEEE Trans. Inf. Technol. Biomed., vol. 15, no. 4, pp. 640–646, Jul. 2011. doi: 10.1109/TITB.2011.2151258 21690019 PMC11977612

[4] Chan H. P., Hadjiiski L. M., and Samala R. K., ‘‘Computer-aided diagnosis in the era of deep learning,” Med. Phys., vol. 47, no. 5, pp. e218–e227, 2020. doi: 10.1002/mp.13764 32418340 PMC7293164

[5] Mohebian M. R., Marateb H. R., Mansourian M., Mañanas M. A., and Mokarian F., ‘‘A hybrid computer-aided-diagnosis system for prediction of breast cancer recurrence (HPBCR) using optimized ensemble learning,” Comput. Struct. Biotechnol. J., vol. 15, pp. 75–85, Jan. 2017. doi: 10.1016/j.csbj.2016.11.004 28018557 PMC5173316

[6] Sajjad M., Khan S., Shoaib M., Ali H., Jan Z., Muhammad K., et al., ‘‘Computer aided system for leukocytes classification and segmentation in blood smear images,” in Proc. Int. Conf. Frontiers Inf.Technol. (FIT), Dec. 2016, pp. 99–104.

[7] Raja N. S. M., Fernandes S. L., Dey N., Satapathy S. C., and Rajinikanth V., ‘‘Contrast enhanced medical MRI evaluation using tsallis entropy and region growing segmentation,” J. Ambient Intell. Humanized Comput., vol. 2018, pp. 1–12, May 2018.

[8] Duan Y., Wang J., Hu M., Zhou M., Li Q., Sun L., et al., ‘‘Leukocyte classification based on spatial and spectral features of microscopic hyperspectral images,” Opt. Laser Technol., vol. 112, pp. 530–538, Apr. 2019.

[9] Cao H., Liu H., and Song E., ‘‘A novel algorithm for segmentation of leukocytes in peripheral blood,” Biomed. Signal Process. Control, vol. 45, pp. 10–21, Aug. 2018.

[10] Abdulhay E., Mohammed M. A., Ibrahim D. A., Arunkumar N., and Venkatraman V., ‘‘Computer aided solution for automatic segmenting and measurements of blood leucocytes using static microscope images,” J. Med. Syst., vol. 42, no. 4, p. 58, Apr. 2018. doi: 10.1007/s10916-018-0912-y 29455440

[11] Wang L., Yang S., Yang S., Zhao C., Tian G., Gao Y., Chen Y., et al., ‘‘Automatic thyroid nodule recognition and diagnosis in ultrasound imaging with the YOLOv2 neural network,” World J. Surg. Oncol., vol. 17, no. 1, pp. 1–9, Dec. 2019. 30621704 10.1186/s12957-019-1558-z PMC6325802

[12] Hegde R. B., Prasad K., Hebbar H., and Singh B. M. K., ‘‘Comparison of traditional image processing and deep learning approaches for classification of WBCs in peripheral blood smear images,” Biocyber. Biomed. Eng., vol. 39, no. 2, pp. 382–392, Apr. 2019.

[13] Sajjad M., Khan S., Muhammad K., Wu W., Ullah A., and Baik S. W., ‘‘Multi-grade brain tumor classification using deep CNN with extensive data augmentation,” J. Comput. Sci., vol. 30, pp. 174–182, Jan. 2019.

[14] Kutlu H., Avci E., and Özyurt F., ‘‘WBCs detection and classification based on regional convolutional neural networks,” Med. Hypotheses, vol. 135, Feb. 2020, Art. no. 109472. 10.1016/j.mehy.2019.109472 31760248

[15] Sajjad M., Khan S., Jan Z., Muhammad K., Moon H., Kwak J. T., et al., ‘‘Leukocytes classification and segmentation in microscopic blood smear: A resource-aware healthcare service in smart cities,” IEEE Access, vol. 5, pp. 3475–3489, 2017.

[16] Xing F. and Yang L., ‘‘Robust nucleus/cell detection and segmentation in digital pathology and microscopy images: A comprehensive review,” IEEE Rev. Biomed. Eng., vol. 9, pp. 234–263, Jan. 2016.Toh, Tzen S., Frank Dondelinger, and Dennis Wang. "Looking beyond the hype: applied AI and machine learning in translational medicine." EBioMedicine 47 (2019): 607–615.

[17] https://www.kaggle.com/datasets/paultimothymooney/blood-cells.

[18] Berger ML, Martin BC, Husereau D, Worley K, Allen JD, Yang W, et al. A questionnaire to assess the relevance and credibility of observational studies to inform health care decision making: an ISPOR-AMCP-NPC Good Practice Task Force report. Value in health. 2014 Mar 1;17(2):143–56. doi: 10.1016/j.jval.2013.12.011 24636373 PMC4217656

[19] Rosyadi T., Arif A., Nopriadi B. Achmad, and Faridah, “Classification of leukocyte images using K-Means Clustering based on geometry features,” in 2016 6th International Annual Engineering Seminar (InAES), Yogyakarta, Indonesia, Aug. 2016, pp. 245–249. doi: 10.1109/INAES.2016.7821942

[20] Gautam A., Singh P., Raman B., and Bhadauria H., “Automatic classification of leukocytes using morphological features and Naïve Bayes classifier,” IEEE Reg. 10 Annu. Int. Conf. Proceedings/TENCON, pp. 1023–1027, 2017, doi: 10.1109/TENCON.2016.7848161

[21] Savkare S. S., and Narote S. P., “Blood Cell Segmentation from Microscopic Blood Images,” in 2015 International conference on Information Processing (ICIP), Puna, India, Dec. 2015, pp. 502–505. doi: 10.1109/INFOP.2015.7489435

[22] Toh Tzen S., Dondelinger Frank, and Wang Dennis. "Looking beyond the hype: applied AI and machine learning in translational medicine." EBioMedicine 47 (2019): 607–615. doi: 10.1016/j.ebiom.2019.08.027 31466916 PMC6796516

[23] Tai W. -L, Hu R. -M, Hsiao H. C. W, Chen R. -M and Tsai J. J. P, "Blood Cell Image Classification Based on Hierarchical SVM," 2011 IEEE International Symposium on Multimedia, 2011, pp. 129–136, doi: 10.1109/ISM.2011.29

[24] Şengür, Akbulut Y., Budak Ü and Cömert Z, "White Blood Cell Classification Based on Shape and Deep Features," 2019 International Artificial Intelligence and Data Processing Symposium (IDAP), 2019, pp. 1–4, doi: 10.1109/IDAP.2019.8875945

[25] Elen A. and Turan M. K., “Classifying WBCs using machine learning algorithms,” Uluslar. Muhendis. Arast. Ve Gelistirme Derg., pp. 141–152, 2019.

[26] Huang D.-C. and Hung K.-D., “Leukocyte nucleus segmentation and recognition in color blood-smear images,” in 2012 IEEE International Instrumentation and Measurement Technology Conference Proceedings, Graz, Austria, May 2012, pp. 171–176. doi: 10.1109/I2MTC.2012.6229443

[27] Yampri P., Pintavirooj C., Daochai S., and Teartulakarn S., “White Blood Cell Classification based on the Combination of Eigen Cell and Parametric Feature Detection,” in 2006 1ST IEEE Conference on Industrial Electronics and Applications, Singapore, May 2006, pp. 1–4. doi: 10.1109/ICIEA.2006.257341

[28] Tavakoli Sajad, et al. "New segmentation and feature extraction algorithm for classification of WBCs in peripheral smear images." Scientific Reports 11.1 (2021): 1–13. 10.1038/s41598-021-98599-0 PMC8484470 34593873

[29] Malkawi Areej, et al. "WBCs classification using convolutional neural network hybrid system." 2020 IEEE 5th Middle East and Africa conference on biomedical engineering (MECBME). IEEE, 2020.

[30] Gupta D., Arora J., Agrawal U., Khanna A., de Albuquerque V.H.C., Optimized Binary Bat Algorithm for classification of WBCs, Measurement (2019), 10.1016/j.measurement.2019.01.002.

[31] Lee H. and Chen Y.-P.-P., ‘‘Cell morphology based classification for red cells in blood smear images,” Pattern Recognit. Lett., vol. 49, pp. 155–161, Nov. 2014.

[32] Wibawa Made Satria. "A comparison study between deep learning and conventional machine learning on WBCs classification." 2018 International Conference on Orange Technologies (ICOT). IEEE, 2018.

[33] Toğaçar M., Ergen B., and Cömert Z., “Classification of WBCs using deep features obtained from Convolutional Neural Network models based on the combination of feature selection methods,” Appl. Soft Comput., vol. 97, p. 106810, Dec. 2020.

[34] Baghel N., Verma U. & Nagwanshi K.K. WBCs-Net: type identification of WBCs using convolutional neural network. Multimed Tools Appl (2021). 10.1007/s11042-021-11449-z.

[35] Lippeveld M, Knill C, Ladlow E, Fuller A, Michaelis LJ, Saeys Y, et al. Classification of human WBCs using machine learning for stain‐free imaging flow cytometry. Cytometry Part A. 2020 Mar;97(3):308–19. 10.1002/cyto.a.23920 31688997

[36] Rawat J., Singh A., Bhadauria H., Virmani J., and Devgun J. S., ‘‘Appli- cation of ensemble artificial neural network for the classification of WBCs using microscopic blood images,” Int. J. Comput. Syst. Eng., vol. 4, nos. 2–3, pp. 202–216, 2018.

[37] Nazlibilek S., Karacor D., Ercan T., Sazli M. H., Kalender O., and Ege Y., ‘‘Automatic segmentation, counting, size determination and classification of WBCs,” Measurement, vol. 55, no. 3, pp. 58–65, 2014.

[38] Sadeghian F., Seman Z., Ramli A. R., Kahar B. H. A., and Saripan M.-I., ‘‘A framework for white blood cell segmentation in microscopic blood images using digital image processing,” Biol. Procedures Online, vol. 11, no. 1, p. 196, 2009. doi: 10.1007/s12575-009-9011-2 19517206 PMC3055951

[39] Macawile M. J., Quinones V. V., Ballado A., Cruz J. D., and Caya M. V., “White blood cell classification and counting using convolutional neural network,” in 2018 3rd International Conference on Control and Robotics Engineering (ICCRE), Nagoya, Apr. 2018, pp. 259–263.

[40] Liang G., Hong H., Xie W., and Zheng L., “Combining Convolutional Neural Network with Recursive Neural Network for Blood Cell Image Classification,” IEEE Access, vol. 6, pp. 36188–36197, 2018.

[41] Sharma M., Bhave A., and Janghel R. R., “White Blood Cell Classification Using Convolutional Neural Network,” in Soft Computing and Signal Processing, vol. 900, Wang J, Reddy G. R. M, Prasad V. K, and Reddy V. S, Eds Singapore: Springer Singapore, 2019, pp. 135–143.

[42] Togacar M., Ergen B., and Sertkaya M. E., “Subclass Separation of White Blood Cell Images Using Convolutional Neural Network Models,” Elektron. Ir Elektrotechnika, vol. 25, no. 5, pp. 63–68, Oct. 2019.

[43] Mohamed E. H., El-Behaidy W. H., Khoriba G., and Li J., “Improved WBCs Classification based on Pre-trained Deep Learning Models,” J. Commun. Softw. Syst., vol. 16, no. 1, pp. 37–45, Mar. 2020.

[44] Journal, Yao X., Sun K, Bu X, Zhao C, and Jin Y, “Classification of WBCs using weighted optimize deformable convolutional neural networks convolutional neural networks,” Artif. Cells, Nanomedicine, Biotechnol., vol. 49, no. 1, pp. 147–155, 2021. 10.1080/21691401.2021.1879823 33533656

[45] Khosrosereshki M. A. and Menhaj M. B., ‘‘A fuzzy based classifier for diagnosis of acute lymphoblastic leukemia using blood smear image processing,” in Proc. 5th Iranian Joint Congr. Fuzzy Intell. Syst. (CFIS), Mar. 2017, pp. 13–18.

[46] Bouchet A., Montes S., Ballarin V., and Díaz I., ‘‘Intuitionistic fuzzy set and fuzzy mathematical morphology applied to color leukocytes segmentation,” Signal, Image Video Process., vol. 14, no. 3, pp. 557–564, Apr. 2019.

[47] Jha K. K. and Dutta H. S., ‘‘Mutual information-based hybrid model and deep learning for acute lymphocytic leukemia detection in single cell blood smear images,” Comput. Methods Programs Biomed., vol. 179, Oct. 2019, Art. no. 104987. doi: 10.1016/j.cmpb.2019.104987 31443862

[48] Ullah A, Muhammad K, Hussain T, Baik SW, De Albuquerque VH. Event-oriented 3D convolutional features selection and hash codes generation using PCA for video retrieval. IEEE Access. 2020 Oct 9;8:196529–40.

[49] Sabzi Shahrebabaki A, Imran AS, Olfati N, Svendsen T. A comparative study of deep learning techniques on frame-level speech data classification. Circuits, Systems, and Signal Processing. 2019 Aug 15;38:3501–20.

[50] Kastrati Z, Imran AS, Yayilgan SY. The impact of deep learning on document classification using semantically rich representations. Information Processing & Management. 2019 Sep 1;56(5):1618–32.

[51] Ullah A, Muhammad K, Hussain T, Baik SW. Conflux LSTMs network: A novel approach for multi-view action recognition. Neurocomputing. 2021 May 7;435:321–9.

[52] Banik PP, Saha R, Kim KD. An automatic nucleus segmentation and CNN model-based classification method of white blood cell. Expert Systems with Applications. 2020 Jul 1;149:113211.

[53] Choi JW, Ku Y, Yoo BW, Kim JA, Lee DS, Chai YJ, et al. White blood cell differential count of maturation stages in bone marrow smear using dual-stage convolutional neural networks. PloS one. 2017 Dec 11;12(12):e0189259. doi: 10.1371/journal.pone.0189259 29228051 PMC5724840

[54] Karthikeyan MP, Venkatesan R. Interpolative leishman-stained transformation invariant deep pattern classification for WBCs. Soft Computing. 2020 Aug;24(16):12215–25.

[55] Acevedo A, Alférez S, Merino A, Puigví L, Rodellar J. Recognition of peripheral blood cell images using convolutional neural networks. Computer methods and programs in biomedicine. 2019 Oct 1;180:105020. doi: 10.1016/j.cmpb.2019.105020 31425939

[56] Safuan S. N. M., Md Tomari M. R., and Wan Zakaria W. N., ‘‘White blood cell (WBC) counting analysis in blood smear images using various color segmentation methods,” Measurement, vol. 116, pp. 543–555, Feb. 2018.

[57] Huang D.-C., Hung K.-D., and Chan Y.-K., ‘‘A computer assisted method for leukocyte nucleus segmentation and recognition in blood smear images,” J. Syst. Softw., vol. 85, no. 9, pp. 2104–2118, Sep. 2012.

[58] Danyali H., Helfroush M. S., and Moshavash Z., ‘‘Robust leukocyte segmentation in blood microscopic images based on intuitionistic fuzzy divergence,” in Proc. 22nd Iranian Conf. Biomed. Eng. (ICBME), Nov. 2015, pp. 275–280.

[59] Manik S., Saini L. M., and Vadera N., ‘‘Counting and classification of white blood cell using artificial neural network (ANN),” in Proc. IEEE 1st Int. Conf. Power Electron., Intell. Control Energy Syst. (ICPEICES), Jul. 2016, pp. 1–5.

[60] Li Y., Zhu R., Mi L., Cao Y., and Yao D., ‘‘Segmentation of white blood cell from acute lymphoblastic leukemia images using dual- threshold method,” Comput. Math. Methods Med., vol. 2016, May 2016, Art. no. 9514707. doi: 10.1155/2016/9514707 27313659 PMC4893444

[61] Wang Q., Chang L., Zhou M., Li Q., Liu H., and Guo F., ‘‘A spectral and morphologic method for white blood cell classification,” Opt. Laser Technol., vol. 84, pp. 144–148, Oct. 2016.

[62] Negm A. S., Hassan O. A., and Kandil A. H., ‘‘A decision support system for acute leukaemia classification based on digital microscopic images,” Alexandria Eng. J., vol. 57, no. 4, pp. 2319–2332, Dec. 2018.

[63] Kermani S., Amin M., and Talebi A., ‘‘Recognition of acute lymphoblas- tic leukemia cells in microscopic images using k-means clustering and support vector machine classifier,” J. Med. Signals Sensors, vol. 5, no. 1, p. 49, 2015. PMC4335145 25709941

[64] Agaian S., Madhukar M., and Chronopoulos A. T., ‘‘A new acute leukaemia-automated classification system,” Comput. Methods Biomech. Biomed. Eng., Imag. Visualizat., vol. 6, no. 3, pp. 303–314, May 2018.

[65] Tantikitti S., Tumswadi S., and Premchaiswadi W., ‘‘Image processing for detection of dengue virus based on WBC classification and decision tree,” in Proc. 13th Int. Conf. ICT Knowl. Eng., Nov. 2015, pp. 84–89.

[66] Rawat J., Singh A., Bhadauria H. S., Virmani J., and Devgun J. S., ‘‘Clas- sification of acute lymphoblastic leukaemia using hybrid hierarchical classifiers,” Multimedia Tools Appl., vol. 76, no. 18, pp. 19057–19085, Sep. 2017.

[67] Shaikhina T., Lowe D., Daga S., Briggs D., Higgins R., and Khovanova N., ‘‘Decision tree and random forest models for outcome prediction in antibody incompatible kidney transplantation,” Biomed. Signal Process. Control, vol. 52, pp. 456–462, Jul. 2019. [Online]. Available: https://creativecommons.org/licenses/by/4.0/, doi: 10.1016/j.bspc.2017.01.012

[68] Abdeldaim A. M., Sahlol A. T., Elhoseny M., and Hassanien A. E., ‘‘Computer-aided acute lymphoblastic leukemia diagnosis system based on image analysis,” in AAdvances in Soft Computing and Machine Learn- ing in Image Processing. Springer, 2018, pp. 131–147.

[69] Mathur A., Tripathi A., and Kuse M., ‘‘Scalable system for classification of WBCs from Leishman stained blood stain images,” J. Pathol. Informat., vol. 4, no. 2, p. 15, 2013. 10.4103/2153-3539.109883 PMC3678750 23766937

[70] Hegde R. B., Prasad K., Hebbar H., and Singh B. M. K., ‘‘Development of a robust algorithm for detection of nuclei of WBCs in peripheral blood smear images,” Multimedia Tools Appl., vol. 78, no. 13, pp. 17879–17898, Jul. 2019.

[71] Su M.-C., Cheng C.-Y., and Wang P.-C., ‘‘A neural-network-based approach to white blood cell classification,” Sci. World J., vol. 2014, Jan. 2014, Art. no. 796371. doi: 10.1155/2014/796371 24672374 PMC3929189

[72] Sadeghian F., Seman Z., Ramli A. R., Kahar B. H. A., and Saripan M.-I., ‘‘A framework for white blood cell segmentation in microscopic blood images using digital image processing,” Biol. Procedures Online, vol. 11, no. 1, p. 196, 2009. doi: 10.1007/s12575-009-9011-2 19517206 PMC3055951

[73] Liang G., Hong H., Xie W., and Zheng L., “Combining Convolutional Neural Network with Recursive Neural Network for Blood Cell Image Classification,” IEEE Access, vol. 6, pp. 36188–36197, 2018.

[74] Mohamed E. H., El-Behaidy W. H., Khoriba G., and Li J., “Improved WBCs Classification based on Pre-trained Deep Learning Models,” J. Commun. Softw. Syst., vol. 16, no. 1, pp. 37–45, Mar. 2020.

[75] Journal, Yao X., Sun K, Bu X, Zhao C, and Jin Y, “Classification of WBCs using weighted optimize deformable convolutional neural networks convolutional neural networks,” Artif. Cells, Nanomedicine, Biotechnol., vol. 49, no. 1, pp. 147–155, 2021. 10.1080/21691401.2021.1879823 33533656

[76] Çınar and Tuncer S. A., “Classification of lymphocytes, monocytes, eosinophils, and neutrophils on WBCs using hybrid Alexnet-GoogleNet-SVM,” SN Appl. Sci., vol. 3, no. 4, p. 503, Apr. 2021.

[77] Liu J., Shahroudy A., Perez M., Wang G., Duan L.-Y., and Kot A. C., ‘‘NTU RGB+D 120: A large-scale benchmark for 3D human activity understanding,” IEEE Trans. Pattern Anal. Mach. Intell., vol. 42, no. 10, pp. 2684–2701, Oct. 2019. doi: 10.1109/TPAMI.2019.2916873 31095476

[78] Fan H., Zhang F., Xi L., Li Z., Liu G., and Xu Y., ‘‘LeukocyteMask: An automated localization and segmentation method for leukocyte in blood smear images using deep neural networks,” J. Biophotonics, vol. 12, no. 7, Jul. 2019, Art. no. e201800488. doi: 10.1002/jbio.201800488 30891934

[79] Hegde R. B., Prasad K., Hebbar H., and K Singh B. M., ‘‘Feature extraction using traditional image processing and convolutional neural network methods to classify WBCs: A study,” Australas. Phys. Eng. Sci. Med., vol. 42, no. 2, pp. 627–638, Jun. 2019. 30830652 10.1007/s13246-019-00742-9

[80] Acevedo A., Alférez S., Merino A., Puigví L., and Rodellar J., ‘‘Recog- nition of peripheral blood cell images using convolutional neural net- works,” Comput. Methods Programs Biomed., vol. 180, Oct. 2019, Art. no. 105020. 10.1016/j.cmpb.2019.105020 31425939

[81] Qin F., Gao N., Peng Y., Wu Z., Shen S., and Grudtsin A., ‘‘Fine-grained leukocyte classification with deep residual learning for microscopic images,” Comput. Methods Programs Biomed., vol. 162, pp. 243–252, Aug. 2018. doi: 10.1016/j.cmpb.2018.05.024 29903491

[82] Tiwari P., Qian J., Li Q., Wang B., Gupta D., Khanna A., Rodrigues J. J. P. C., and de Albuquerque V. H. C., ‘‘Detection of subtype blood cells using deep learning,” Cognit. Syst. Res., vol. 52, pp. 1036–1044, Dec. 2018.

[83] Hung J. and Carpenter A., ‘‘Applying faster R-CNN for object detection on malaria images,” in Proc. IEEE Conf. Comput. Vis. Pattern Recognit. Workshops, vol. 2017, pp. 56–61. doi: 10.1109/cvprw.2017.112 34938593 PMC8691760

[84] Razzak M. I. and Naz S., ‘‘Microscopic blood smear segmentation and classification using deep contour aware CNN and extreme machine learn- ing,” in Proc. IEEE Conf. Comput. Vis. Pattern Recognit. Workshops (CVPRW), Jul. 2017, pp. 49–55.

[85] Vogado L. H. S., Veras R. M. S., Araujo F. H. D., Silva R. R. V., and Aires K. R. T., ‘‘Leukemia diagnosis in blood slides using transfer learning in CNNs and SVM for classification,” Eng. Appl. Artif. Intell., vol. 72, pp. 415–422, Jun. 2018.

[86] Habibzadeh M., Jannesari M., Rezaei Z., Baharvand H., and Totonchi M., ‘‘Automatic white blood cell classification using pre-trained deep learn- ing models: Resnet and inception,” in Proc. 10th Int. Conf. Mach. Vis. (ICMV), 2018, vol. 10696, Art. no. 1069612.

[87] Song Y., Zhang L., Chen S., Ni D., Li B., Zhou Y., Lei B., and Wang T., ‘‘A deep learning based framework for accurate segmentation of cervical cytoplasm and nuclei,” in Proc. 36th Annu. Int. Conf. IEEE Eng. Med. Biol. Soc., Aug. 2014, pp. 2903–2906. 10.1109/EMBC.2014.6944230 25570598

[88] Özyurt F., ‘‘A fused CNN model for WBC detection with MRMR fea- ture selection and extreme learning machine,” Soft Comput., vol. 24, pp. 8163–8172, Jun. 2019.

[89] Rehman A., Abbas N., Saba T., Rahman S. I. U., Mehmood Z., and Kolivand H., ‘‘Classification of acute lymphoblastic leukemia using deep learning,” Microsc. Res. Techn., vol. 81, no. 11, pp. 1310–1317, Nov. 2018. doi: 10.1002/jemt.23139 30351463

[90] Bani-Hani D., Khan N., Alsultan F., Karanjkar S., and Nagarur N., ‘‘Clas- sification of leucocytes using convolutional neural network optimized through genetic algorithm,” in Proc. 7th Annu. World Conf. Soc. Ind. Syst. Eng., Oct. 2018, pp. 1–6.

[91] Di Ruberto C., Loddo A., and Puglisi G., ‘‘Blob detection and deep learning for leukemic blood image analysis,” Appl. Sci., vol. 10, no. 3, p. 1176, Feb. 2020.

[92] Loey M., Naman M., and Zayed H., ‘‘Deep transfer learning in diagnosing leukemia in blood cells,” Computers, vol. 9, no. 2, p. 29, Apr. 2020.

[93] Ma L., Shuai R., Ran X., Liu W., and Ye C., ‘‘Combining DC-GAN with ResNet for blood cell image classification,” Med. Biol. Eng. Comput., vol. 4, pp. 1–14, Mar. 2020. doi: 10.1007/s11517-020-02163-3 32221797

[94] Baydilli Y. Y. and Atila Ü., ‘‘Classification of WBCs using capsule networks,” Comput. Med. Imag. Graph., vol. 80, Mar. 2020, Art. no. 101699. 10.1016/j.compmedimag.2020.101699 32000087

[95] Tobias R. R., Carlo De Jesus L., Mital M. E., Lauguico S., Guillermo M., Vicerra R. R., Bandala A., and Dadios E., ‘‘Faster R-CNN model with momentum optimizer for RBC and WBC variants classification,” in Proc. IEEE 2nd Global Conf. Life Sci. Technol. (LifeTech), Mar. 2020, pp. 235–239.

[96] Hosseinzadeh Kassani S., Hosseinzadeh kassani P., Wesolowski M. J., Schneider K. A., and Deters R., ‘‘A hybrid deep learning architecture for leukemic B-lymphoblast classification,” 2019, arXiv:1909.11866. [Online]. Available: http://arxiv.org/abs/1909.11866.

[97] Baghel N., Verma U., and Nagwanshi K. K., “WBCs-Net: type identification of WBCs using convolutional neural network,” Multimed Tools Appl, vol. 81, no. 29, pp. 42131–42147, Dec. 2022, doi: 10.1007/s11042-021-11449-z

[98] Shahzad A., Raza M., Shah J. H., Sharif M., and Nayak R. S., “Categorizing WBCs by utilizing deep features of proposed 4B-AdditionNet-based CNN network with ant colony optimization,” Complex Intell. Syst., vol. 8, no. 4, pp. 3143–3159, Aug. 2022, doi: 10.1007/s40747- 021-00564-x. C.

[99] Cheuque, Querales M., León R, Salas R, and Torres R., “An Efficient Multi-Level Convolutional Neural Network Approach for WBCs Classification,” Diagnostics, vol. 12, no. 2, p. 248, Jan. 2022, doi: 10.3390/diagnostics12020248 35204339 PMC8871319

[100] Hosseini M., Bani-Hani D., and Lam S. S., “Leukocytes Image Classification Using Optimized Convolutional Neural Networks,” Expert Systems with Applications, vol. 205, p. 117672, Nov. 2022, doi: 10.1016/j.eswa.2022.117672

[101] Balasubramanian K., Ananthamoorthy N. P., and Ramya K., “An approach to classify WBCs using convolutional neural network optimized by particle swarm optimization algorithm,” Neural Comput & Applic, vol. 34, no. 18, pp. 16089–16101, Sep. 2022, doi: 10.1007/s00521-022-07279-1

[102] Khalil A. J. and Abu-Naser S. S., “Diagnosis of Blood Cells Using Deep Learning,” vol. 6, no. 2, 2022.

[103] Sharma S. et al., “Deep Learning Model for the Automatic Classification of WBCs,” Computational Intelligence and Neuroscience, vol. 2022, pp. 1–13, Jan. 2022, doi: 10.1155/2022/7384131 35069725 PMC8769872

[104] Razzak M. I., Naz S., and Zaib A., ‘‘Deep learning for medical image pro- cessing: Overview, challenges and the future,” in *Classification BioApps*. Ithaca, NY, USA: Cornell Univ., 2018, pp. 323–350.

[105] Altaf F., Islam S. M. S., Akhtar N., and Janjua N. K., ‘‘Going deep in medical image analysis: Concepts, methods, challenges, and future directions,” *IEEE Access*, vol. 7, pp. 99540–99572, 2019.

[106] Muhammad K., Hussain T., Del Ser J., Palade V., and de Albuquerque V. H. C., ‘‘DeepReS: A deep learning-based video summarization strategy for resource-constrained industrial surveillance scenarios,” *IEEE Trans*. *Ind*. *Informat*., vol. 16, no. 9, pp. 5938–5947, Sep. 2020.

[107] Hussain T., Muhammad K., Ullah A., Del Ser J., Gandomi A. H., Sajjad M., et al, ‘‘Multi-view sum- marization and activity recognition meet edge computing in IoT envi- ronments,” *IEEE Internet Things J*., early access, Sep. 29, 2020, doi: 10.1109/JIOT.2020.3027483

[108] Han T., Muhammad K., Hussain T., Lloret J., and Baik S. W., ‘‘An efficient deep learning framework for intelligent energy management in IoT networks,” *IEEE Internet Things J*., early access, Aug. 3, 2020, doi: 10.1109/JIOT.2020.3013306

[109] Muhammad K., Hussain T., and Baik S. L., ‘‘Efficient CNN based summarization of surveillance videos for resource-constrained devices,” *Pattern Recognit*. *Lett*., vol. 130, pp. 370–375, Feb. 2020.

[110] Zhang C., Bengio S., Hardt M., Recht B., and Vinyals O., ‘‘Under- standing deep learning requires rethinking generalization,” 2016, *arXiv*:*1611*.*03530*. [Online]. Available: http://arxiv.org/abs/1611.03530

[111] Mehmood I., Hussain T., Hassan I., Rho S., and Sajjad M., ‘‘Let the deaf understand: Mainstreaming the marginalized in context with personalized digital media services and social needs,” in *Proc*. *IEEE Int*. *Conf*. *Multi- media Expo Workshops (ICMEW)*, Jul. 2017, pp. 220–225.

[112] Erickson B. J., Korfiatis P., Akkus Z., Kline T., and Philbrick K., ‘‘Toolk- its and libraries for deep learning,” *J*. *Digit*. *Imag*., vol. 30, no. 4, pp. 400–405, Aug. 2017. 10.1007/s10278-017-9965-6 PMC5537091 28315069

[113] Muhammad K., Hussain T., Tanveer M., Sannino G., and de Albuquerque V. H. C., ‘‘Cost-effective video summarization using deep CNN with hierarchical weighted fusion for IoT surveillance networks,” *IEEE Internet Things J*., vol. 7, no. 5, May 2020, doi: 10.1109/JIOT.2019.2950469

[114] Marzahl C., Aubreville M., Voigt J., and Maier A., ‘‘Classification of leukemic b-lymphoblast cells from blood smear microscopic images with an attention-based deep learning method and advanced augmenta- tion techniques,” in *ISBI C-NMC Challenge*: *Classification Cancer Cell Imaging*. Springer, 2019, pp. 13–22.

[115] Huang P., Wang J., Zhang J., Shen Y., Liu C., Song W., et al., ‘‘Attention-aware residual network based manifold learning for WBCs classification,” *IEEE J*. *Biomed*. *Health Informat*., early access, Jul. 29, 2020, doi: 10.1109/JBHI.2020.3012711 32750980

[116] Hussain M.A., Ibtihaj A., Shaukat A., and Islam Z.U. "Leukocytes segmentation and classification in digital microscopic images." In 2021 4th International Conference on Computing & Information Sciences (ICCIS), pp. 1–6. IEEE, 2021.

[117] Ahmad R., Awais M., Kausar N., and Akram T., “WBCs classification using entropy-controlled deep features optimization,” Diagnostics (Basel), vol. 13, no. 3, p. 352, 2023. 36766457 10.3390/diagnostics13030352 PMC9914384

[118] Bhuiyan M, Islam MS. A new ensemble learning approach to detect malaria from microscopic red blood cell images. Sensors International. 2023 Jan 1;4:100209.

[119] Elhassan T. A. et al., “Classification of atypical WBCs in acute myeloid leukemia using a two-stage hybrid model based on deep convolutional autoencoder and deep convolutional neural network,” Diagnostics (Basel), vol. 13, no. 2, p. 196, 2023. 36673006 10.3390/diagnostics13020196 PMC9858290

[120] Kakumani AK, Katla V, Rekhawar V, Yellakonda AR. A Comparative Analysis for Leukocyte Classification Based on Various Deep Learning Models Using Transfer Learning. In2023 4th International Conference for Emerging Technology (INCET) 2023 May 26 (pp. 1–6). IEEE.

[121] Loizidou K., Elia R., Pitris C. Computer-aided breast cancer detection and classification in mammography: A comprehensive review. Computers in Biology and Medicine. Volume 153, 2023. 10.1016/j.compbiomed.2023.106554 36646021

[122] Abou Ali M., Dornaika F., Arganda-Carreras I. White Blood Cell Classification: Convolutional Neural Network (CNN) and Vision Transformer (ViT) under Medical Microscope. Algorithms 2023, 16, 525.

[123] Saidani O., Umer M., Alturki N. White blood cells classification using multi-fold pre-processing and optimized CNN model. Sci Rep 14, 3570 (2024). 38347011 10.1038/s41598-024-52880-0 PMC10861568

[124] Zhu Z, Wang SH, Zhang YD. ReRNet: A Deep Learning Network for Classifying Blood Cells. Technol Cancer Res Treat. 2023 Jan-Dec;22:15330338231165856. doi: 10.1177/15330338231165856 PMC10061646 36977533

