# Expression of Concern: Classification of white blood cells (leucocytes) from blood smear imagery using machine and deep learning models: A global scoping review

**DOI:** 10.1371/journal.pone.0353227

**Published:** 2026-07-07

**Authors:** The PLOS One Editors

After this article [1] was published, the following concerns were noted:

- The article cited as Reference 103 was retracted before [1] was published.
- The article cited as Reference 54 was retracted after [1] was published.
- The articles cited as References 49, 50, 51, and 111 do not appear to support the corresponding cited statements [1].
- The articles cited as References 113, 114, 115, and 116 do not appear to be cited in the article.

The corresponding author responded that the authors were unaware of the retracted status of Reference 103 prior to this article’s publication. Additionally, the authors stated that References 49, 50, 51, and 111 were cited for the purpose of describing relevant machine learning and deep learning architectures outside of the confines of white blood cell classification, and were not intended to be cited specifically in the context of this application. However, the Editors do not consider this matter to be fully resolved.

In light of the cumulative issues, the *PLOS One* Editors issue this Expression of Concern. Readers are advised to interpret the article [1] with caution.

The corresponding author stated that References 113–116 were unintentionally missing from the published article, and has provided the correct locations for these citations below.

In the 4. 1 Lack of publicly accessible datasets subsection of the 4. Limitations of previous studies and future challenges section, Reference 113 is omitted from the fifth sentence of the paragraph.

The correct sentence is: When data are readily available in large quantities, just like in other fields such as environmental science, weather forecasting, and bioinformatics, the issue becomes more relevant for research (e.g., video summarization [106, 113], IoT [107, 113], energy management [108], and so on).

In the i. Data augmentation methods to complete the dataset deficit subsection of the 5. Future research directions section, Reference 114 is omitted from the second sentence of the paragraph.

The correct sentence is: We present data augmentation approach [114] and leverage transfer learning algorithms to enhance the identification of WBCs.

In the 3.2 White blood cell classification using deep learning techniques subsection of the 3. Review of identified relevant literature section, Reference 115 is omitted from the sixth sentence of the fourteenth paragraph.

The correct sentence is: Deep learning models have represented a significant breakthrough in myriad domains and as shown in the identified literature, the use of traditional machine learning models within biomedical applications in general, and WBC classification in particular is undoubtedly shifting toward the use of deep learning models based on dataset size [115].

In the iv. Models for the detection and classification of leukocytes subsection of the 5. Future research directions section, Reference 116 is omitted from the fourth sentence of the paragraph.

The correct sentence is: WBC detection and categorization in images can also be accomplished using a variety of end-to-end designs [116, 122–124].
