# Supplementary material for: Classification of white blood cells (leucocytes) from blood smear imagery using machine and deep learning models: A global scoping review
Source: PLoS One. 2024 Jun 17;19(6):e0292026. doi: 10.1371/journal.pone.0292026 (PMC11182552; doi:10.1371/journal.pone.0292026)
Supplement: S1 Checklist — (DOCX) [file pone.0292026.s001.docx]

**Identification of studies via databases and registers**

Records removed before screening:

Duplicate records removed

(n = 1540)

Records identified from databases (from 2006 to 2023; peer-reviewed; English) *:

Google scholar (n = 1240); Scopus (n = 1460); Web of Science (n = 1050)

**Identification**

Records screened

(n = 2210)

Records excluded**

(n = 1167)

Reports sought for retrieval

(n = 1043)

Reports not retrieved

(n = 608)

**Screening**

Reports assessed for eligibility

(n = 435)

Reports excluded:

Irrelevant abstract and titles (n = 235)

Ineligible blood cell datasets (n = 14)

Shorter than 5 months (n = 50).

Studies included in review

(n = 136)

**Included**
